# Fluctuations of viti- and oleiculture traditions in the Bronze and Iron Age Levant

**DOI:** 10.1371/journal.pone.0330032

**Published:** 2025-09-17

**Authors:** Simone Riehl, Katleen Deckers, Israel Hinojosa-Baliño, Darren R. Gröcke, Dan Lawrence

**Affiliations:** 1 Institute for Archaeological Sciences, University of Tübingen, Tübingen, Germany; 2 Senckenberg Center for Human Evolution and Palaeoenvironment (HEP), University of Tübingen, Tübingen, Germany; 3 Department of Archaeology, Durham University, Durham, United Kingdom; 4 Department of Earth Sciences, Durham University, Durham, United Kingdom; Israel Antiquities Authority, ISRAEL

## Abstract

Various researchers have demonstrated periods of instability in the cultivation of olives and grapes in the eastern Mediterranean, dating back at least to the Early Bronze Age. So far, pollen-based studies have focused primarily on olive cultivation in the southern Levant. Our research extends these studies to include both cash crops throughout the Levant and northern Mesopotamia, including several different climatic zones, to better understand the diversity of human strategies for maintaining agricultural stability. We analysed 1,514 charred olive (*Olea europaea*) and grape (*Vitis vinfera*) seed and wood samples from archaeological sites for their stable carbon isotope ratios to reconstruct Bronze and Iron Age growing conditions. The results, with generally 3.7‰ higher Δ^13^C values in grapevine than in olive, are consistent with the physiological characteristics of the two species, i.e., their water use efficiency, and with their different agronomic needs. Furthermore, higher values in charcoals than in fruits indicate the natural differences in the budgets of water availability associated with the period of formation of the measured plant tissue. Canonical correspondence analysis (CCA) of the complete data set shows clear correlation of the mean Δ^13^C values with reconstructed average precipitation (RAP) and the general north-south and west-east decline in precipitation of the region, as well as with chronology, including a gradual drying trend through time. Principal component analysis (PCA) based on these variables shows a highly diversified relationship between mean and maximum stress levels and RAP at different sites over time. An important trend is the significant accumulation of Iron Age sites in olive-growing and wine-producing regions above 500 mm RAP. However, there are considerable diachronic differences in the stress signals for the two tree crops. Interpolations of the mean Δ^13^C values of the crop species are in good agreement with the layout of the isohyets and visualize the PCA patterns of stable carbon isotope and precipitation relationships thereby confirming the major trends such as a better water availability in the Iron Age. The well-known major climatic fluctuations at 4.2 and 3.2 kyr BP correlate with likely irrigation of olive trees, while there are also drought patterns indicated in the Δ^13^C values at the end of the Middle Bronze Age. In general, the greater commitment to the establishment of agricultural niches and successful production for viticulture compared to oleiculture, which has already been observed in historical times, is confirmed, at least since the Middle Bronze Age and especially in the Iron Age.

## Introduction

Archaeological, historical, and paleoenvironmental evidence in recent decades demonstrated the cultural significance of vine and olive cultivation in human societies [1–11]. Viticulture and olive growing had a profound imprint on human cultural history, shaping landscapes, economies, traditions, and lifestyles in regions around the world [12]. As dietary staples, grapes and olives provided essential nutrients, flavor, and calories to consumers, supplementing other food sources such as cereals, legumes, and animal products [13]. Their cultural significance is partially reflected in their symbolic and ceremonial use in religious practices and offerings, and the ownership of vineyards and olive groves conferred social status and prestige upon members of society in the Bronze and Iron Age Levant [14,15]. During this period both crops were important sources of income for farmers and merchants [16]. Wine and olive oil were exported to neighboring regions and distant markets through complex trade networks, contributing to the prosperity of urban centers and commercial hubs [17–19]. Wine and olive production spurred technological innovations and advances in agricultural practices and storage techniques. Wine and olive presses, built or worked into the bedrock, shaped ancient settlements and landscapes, as did the terraced hillsides for maintaining orchards and vineyards [20–22]. The decline of urban centers, which owed at least part of their wealth and influence to the production and trade of wine and olive oil, is sometimes attributed to Holocene climate dynamics and their presumed influence on agriculture, including grape and olive cultivation [11,23,24]. Palynological and archaeological works, mainly for the Southern Levant region combine climatic and geopolitical aspects to explain variations in olive pollen [25–26].

Stable carbon isotope ratios (δ^13^C) in plant remains can indicate the availability of moisture for plants at a given time and place [27,28]. As such, the values can indicate periods of drought [29,30] and, if additional data are included, irrigation practices [31,32].

In this study we use Δ^13^C values of grape and olive crops to identify the water stress levels of the crops and thus to detect likely local variations in potential biomass productivity levels. We regard this as a measure of the potential exploitability of grapevine and olives, which in turn informs on their economic potential for surplus production and trade. Our working hypothesis is that high Δ^13^C values are associated with high potential biomass productivity levels, which may indirectly provide indication of possible surplus, and therefore volume available to trade, while low Δ^13^C values indicate the opposite. Furthermore, the Δ^13^C values of fruit remains that ripen within a relatively short season may differ from the vegetative tissues of the wood that develop throughout the year, thus capturing different levels of moisture availability.

In addition to regional differences in precipitation – which we include in our analysis in the form of reconstructed modeled values – and their long-term dynamics in the context of climate variability, technologies (e.g., irrigation) are also important and are subject to temporal dynamics.

Archaeological isoscapes based on stable carbon isotopes in archaeobotanical remains of olives and grapes, complementing existing datasets for other crop species (e.g., cereals and legumes), can help researchers to build a more comprehensive picture of the potential productivity of early agricultural systems, regionally different environmental adaptations such as irrigation, and socio-economic dynamics.

By interpolating our local Δ^13^C values, we gain an understanding of past human-environment relationships through the major determinants of agricultural production. In particular, we use our interpolations to reflect the history of the development of viticulture and olive-growing in the context of climate change and agricultural management in relatively broad chronological units.

## Materials and methods

**Fig 1:**
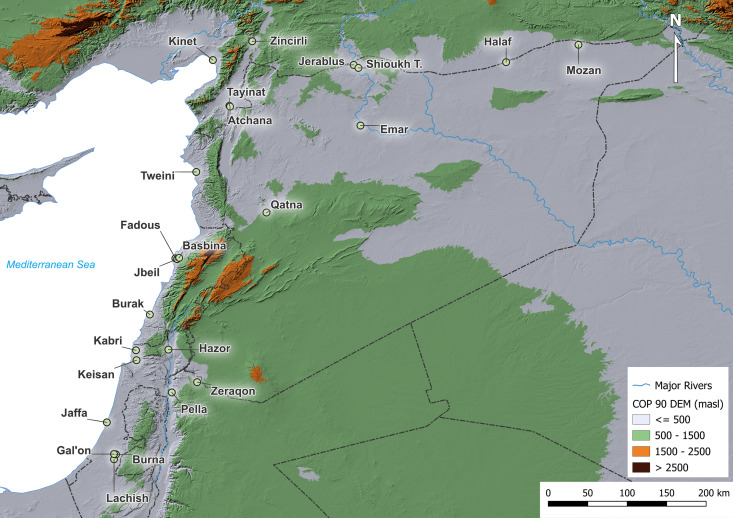
Map of sampling locations of archaeobotanical olive and grape specimens. Digital Elevation Model produced using Copernicus WorldDEM-30 © DLR **e.**V. 2010-2014 and © Airbus Defence and Space GmbH 2014-2018 provided under COPERNICUS by the European Union and ESA; all rights reserved, available at https://doi.org/10.5270/ESA-c5d3d65. Vector data provided by Natural Earth.

For this study, a total of 1,514 charred archaeobotanical remains of olive and grape seeds and wood charcoal from various archaeological sites were measured for their stable carbon isotope ratios (SI stable isotope data.xlsx). Collaborations with excavation projects allowed archaeobotanical sampling and include the sites of Jaffa, Tel Burna [33,34], Tel Hazor, Tel Kabri [31,35], Tel Keisan, and Tel Lachish [36] in Israel, Hirbet ez-Zeraqon [37,38] in Jordan, Baalbek [39], Tell el-Burak [40], and Tell Fadous-Kfarabida [41–43] in Lebanon, Tell Bazi, Emar [44,45], Qatna [46], Tell Halaf [47], Tell Mozan [48], and Tell Tweini [10], and Tell Jerablus [49] in Syria, Kinet Höyük [50], Tell Shioukh-Tahtani (Deckers, unpublished data), Tell Atchana [51], Tell Tayinat, and Zincirli [52] in Türkiye (Fig 1). We obtained permission from the respective excavators to determine and perform isotopic analysis of the archaeobotanical materials from the individual sites.

### Stable carbon isotope analysis

Stable carbon isotope analysis of archaeobotanical remains has become a standard method to analyze the water status of plants during the formation of the measured plant part and thus to reconstruct ancient growing conditions of plants in a broad sense [53–56]. The underlying mechanisms in plants are part of photosynthesis and have been recognized at least since the 1980s [57–59]. The ratio of ^12^C/^13^C in the fixed carbon of the chloroplasts indicates the extent to which the plant was forced to close its stomata due to increased evaporation and to use predominantly the heavier ^13^C to maintain photosynthesis. This mechanism is also called drought stress.

Stable carbon isotope measurements on 923 fruit stones and seeds, including olive stones (n = 558) and grape pips (n = 365) and 410 wood charcoal samples of olive (n = 306) and grape (n = 104) were carried out at the Stable Isotope Biogeochemistry Laboratory (SIBL) at Durham University. We also included 152 measurements of grape pips (n = 65) and olive stones (n = 87) from previous collaborations at Tell Tweini (project at the University of Leuven; published in [10]; analyzed at the Max Planck Institute for Evolutionary Anthropology in Leipzig) and 29 measurements from other previous projects (measurements carried out at the Isotope Geochemistry Laboratory, University of Tübingen; (for more information see also SI_methods.pdf).

We did not apply any correction factors to the raw data, such as subtracting 0.1‰ from measurements in cereals and legumes [60] to compensate for possible charring effects. Instead, we followed the recent study by Ehrlich et al. [61], which found no significant difference between fresh and charred modern olive stones at 250°C. However, a study by Mouraux et al. [62] shows that carbon stable isotope values from pine and oak wood charred at temperatures below 400°C can be incomparable with data from higher charring temperatures, but the possible impact of stable carbon isotopic values from measurements with low C percentages was insignificant in our analyses.

To compare plant isotopic results from different time periods, fluctuations in atmospheric CO_2_ concentrations (δ^13^C_air_) over time must be considered. To account for this, the δ^13^C measurements were transformed into Δ^13^C values using the AIRCO2_LOESS data application, which is based on CO_2_ [63,64].

### Data analysis

The data analysis was conducted with two distinct objectives: (1) to examine the spatial and temporal variations in the Δ^13^C values of grapes and olives, and (2) to ascertain the influencing factors associated with the outcomes of (1). The fundamental premise is that Δ^13^C serves as a proxy for water availability. However, the extent of rainfall during plant tissue formation and the role of irrigation remains unknown.

For precipitation variables, we chose to use projected values rather than modern annual mean precipitation to compensate for temporal changes. No further attempts were made to refine annual or occasional rainfall variability. This was primarily due to major research gaps in these parameters across the entire Fertile Crescent region, and secondly because the analytical setting was based on chronological ranges at the decadal level. We used the model described in [65], which calculated precipitation using modern distributions and hindcast data from Soreq Cave. To link the periods to the precipitation data, we calculated the midpoint of the period dates and then the mean of the available values for precipitation in a 100-year window either side of this midpoint (S3 File). Past Reconstructed Average Precipitation (RAP) was included as an explanatory variable in the multivariate statistical application.

Apart from basic mean and range calculation of the measured Δ^13^C values, summarized in box plots (S1–S3 Figs), multivariate statistics were applied for pattern searching based on chronology and reconstructed mean precipitation. Prior to the multivariate statistical pattern search, some significance tests were performed on the data set (see S1 File for full description of statistical analysis).

### Interpolation

We used interpolation to model the likely layout of isotopic values between sites. By interpolating the local mean Δ^13^C values in large chronological units we aim to visualize the development of grape and olive cultivation in the context of fluctuating water availability. In order to avoid regional differences in absolute chronology and phase designation, and in view of the fact that the earliest of our investigated sites is dated to 3070 BC, we have categorised our time periods in the following sequence: Early Bronze Age (EBA) to the transition of EBA to Middle Bronze Age (MBA), (ca. 3000-ca. 2000 BC); MBA to the transition of MBA to Late Bronze Age (LBA) (ca. 2000-ca. 1500 BC); LBA to the transition of LBA to Iron Age (IA) (ca. 1500-ca. 1200 BC); and Iron Age (ca. 1200-ca. 550 BC).

Since the individual sites with the available samples fall with varying degrees of accuracy into an absolute chronological framework, and since the number of sites that fall together into a short-term chronological framework was small, we have combined several sites into larger chronological time periods of several hundred years. This way, (1) minor chronological inaccuracies (in a dimension of 100 years) of the individual samples could be compensated and (2) the number of localities necessary to increase the density of survey points for a realistic interpolation could be obtained, thus showing long-term developments in water availability for the fruit trees. The combination of this relatively coarse background pattern of cultivation and partially higher resolved time units, as in our PCAs, provides a more accurate picture of cultivation conditions, assuming that variation within periods is fairly limited.

To estimate values for Δ^13^C at a location for which there is no measured value, interpolation uses mathematical functions that predict values from a limited number of sample data points. Three different interpolation methods are used, namely inverse distance weighting (IDW), splines, and kriging. Each method has its own characteristics, strengths, and weaknesses. Interpolation was done in R using the gstat, terra, and mgcv packages (details see S1 File).

In addition to olive and grape, we also compare the diachronic isoscapes with those produced by mean Δ^13^C values for barley. Barley, with its short life cycle and relatively low irrigation requirements, can serve as a more realistic background pattern for the other two crops. The data set for barley is significantly larger and covers many more locations (n = 1005 and 41 ancient locations), which enables reliable interpolation. The dataset of barley was already published [28], but the dataset of grape and olive is new, previously unpublished data. For the wood interpolation, we show only olive, because Δ^13^C values for grape are present from fewer than three sites in some time ranges, meaning interpolation is impossible.

## Results

### General patterns of stable carbon isotope data of olive and grape seeds and wood charcoal

**Fig 2:**
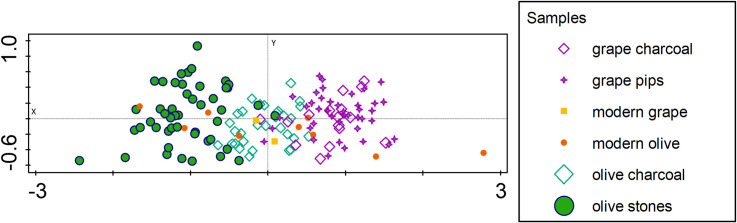
PCA analysis using mean, minimum and maximum Δ^13^C values of grape and olive seeds and charcoal as functional traits.

A comparison of the Δ^13^C values of *Vitis* and *Olea* seeds and charcoal from different locations reveals certain general characteristics (Fig 2 and S1 Fig). The mean value of wood charcoal of olive was found to be 2.1 ± 0.59 ‰ higher than that of olive stones. Given that Δ^13^C values typically reflect water availability during the formation of the measured plant tissue, it is evident that there must be a consistent seasonal difference when comparing wood charcoal from single tree rings and fruits, which form over a much shorter time period. Researchers discovered that the cumulative δ¹³C signals of wood species, including olive and grape, not only reflect environmental conditions throughout the growing season but also the signals stored during previous seasons [66,67]. Consequently, they provide longer-term signals, if not sampled per growth ring [68–71].

In our modern plant samples, leaves, which form over a longer time period than the fruits, tend to have higher values than stones, supporting the general observation of higher stress during the short, comparatively dry season of stone formation. For pulp, there appear to be other mechanisms during fractionation (S2 Fig).

There is a consistent difference in the Δ^13^C values between olive and grape (S1 Fig), which is characterized by an average 3.7‰ higher Δ^13^C value in grape compared to olive over time and space. The PCA of Δ^13^C minima, maxima and means for olive and grape also reflects this species-specific physiological difference in carbon fractionation during photosynthesis within their different time ranges of tissue formation (Fig 2). Inter-species differences appear to be more pronounced for the measured fruit stones than for the wood charcoal, reflecting the signal of year-round water uptake in wood, mainly from precipitation, versus seasonal water availability patterns in fruit. While the Δ^13^C values of wood charcoal from olive trees and grapes show greater similarity, probably due to a closer association with the ecological background pattern of annual precipitation, the seeds of olive trees and grapes differ due to differences in seasonal moisture availability, at least partially due to different months of fruit formation with a shorter fruiting time of grape, differing localities of cultivation and also due to different treatment with respect to irrigation.

Wood and fruit Δ^13^C of the same species are more similar in grape than in olive, suggesting that moisture availability for grape was relatively stable throughout the year, probably due to irrigation of the plants during the drier months. Olive, on the other hand, seems to show a higher differentiation between fruit and charcoal, reflecting very different conditions between annual rainfall and moisture availability at the time of fruit formation, the latter corresponding to the dry season. These differences suggest that vineyards were irrigated more frequently than olive groves (see below).

The modern values for olives seem to be inconsistent compared to the ancient values and are spread over the whole graph (Fig 2 and S2 File), even if the value for olive pulp from the location of Jbeil is subtracted at the far-right corner. At present, we can only speculate about the divergence between modern and ancient values, but we can assume that the sampling in the field includes fruit from feral trees, which received less or no care from modern olive growers. The modern data are therefore removed from the data set in the following analyses.

The natural ranges of stable carbon isotopes in grapevines, as determined by the physiology of the species and cultivars, are not available at present. The few studies published on modern examples from agronomic contexts describe plants grown under intensive management, including irrigation and fertilization. However, since there is also a more or less consistent difference in our ancient mean Δ^13^C values in grape compared to olive (S1 and S2 Figs), it can be assumed that there is a physiological difference in water use efficiency between the two species, as is known, for example, for barley and wheat [27,72].

A study by Ehrlich et al. [61] suggests that the Δ^13^C threshold for olive fruit under severe drought stress is below 15.5 ± 0.5‰. Based on this value, it can be assumed that the Δ^13^C threshold for grape fruit under drought stress is correspondingly higher. Our data yielded Δ^13^C ranges of ancient olive stones between 12.7‰ and 19.8‰, with a mean at 16.2‰, the Δ^13^C ranges of ancient grape pips have ranges between 16.6‰ and 23.7‰, with a mean at 19.9‰, i.e., the mean Δ^13^C range of grape is generally 3.7‰ higher than that of olive. Applying a 3.7‰ higher threshold for grape, i.e., a threshold of 19.2‰, our data would indicate that drought stress could have affected grape yields at Tel Lachish, the earliest Early Bronze Age settlement phase at Hirbet-ez Zeraqon, a brief Middle Bronze Age settlement phase at Tell Tweini around 1800 BC, the mid Early Bronze Age phase at Tell Mozan, and to some extent Qatna and Jaffa at the end of the Late Bronze Age and Tell Tayinat towards the end of the Early Bronze Age. The fact that we do not get a pattern with generally very high or no drought stress at all for olive could be seen as further confirmation that we are correct in our assumptions regarding the assumed threshold for grapes.

It should be noted, however, that the grapes harvested usually contain fully developed fruits. This is particularly the case for the direct consumption of the fresh fruit, which has not been crushed for wine production and the remains of which are usually found in the archaeological record. It can be assumed that water-deficient or underdeveloped fruits most likely remained on the vines and were therefore not frequently deposited in the archaeological context. For the time being, Δ^13^C values of grape seeds from different sites should only be compared relative to each other, rather than classified according to their absolute water availability, until an experimentally determined threshold value is published.

**Fig 3:**
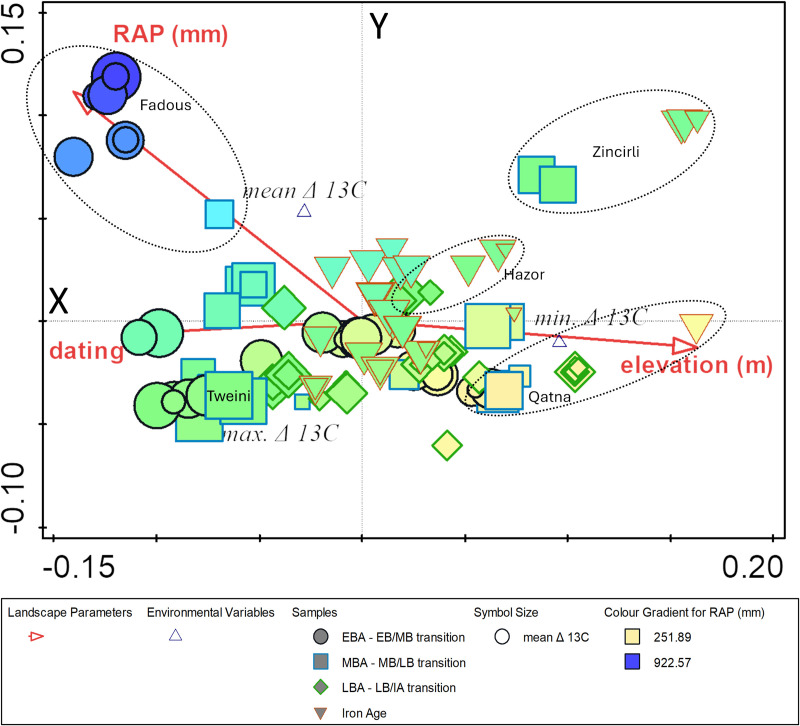
CCA on the complete dataset of olive and grape seeds and wood charcoal not further differentiated using Δ^13^C minima, mean and maxima as response variables and chronology, elevation and RAP as explanatory variables. CCA shows mostly a geographic pattern, i.e., values for specific locations are plotted together and also strongly relate to RAP.

CCA on the complete data of ancient grape and olive, wood and seeds with the response variables of mean, minimum and maximum Δ^13^C values and the explanatory variables of chronology, RAP and elevation provide an overall general pattern with an explained fitted cumulative variation for the first axis of 88% and 100% for the second axis (Fig 3).

In Fig 3 the explanatory parameters (red arrows) each point in the direction of the steepest increase in their values. The angle between the arrows indicates the correlation between each landscape parameter, i.e., while RAP and chronology are correlated, there seems to be no correlation of these two parameters with elevation. This pattern can be explained by the fact that the elevation of a site remains the same throughout time, whereas chronology and RAP occur in different combinations at each site. The calculation process of RAP is related to chronology; however, it also reflects the real correlation of the gradual drying trend over time. This contributes to the reasons why samples from one site are plotted next to each other. Elevation is generally an important landscape parameter explaining the cold sensitivity of grapevine and olive, since temperature generally decreases with increasing elevation (lapse rate at about 6.7°C/km [73] and lower temperatures slow down metabolic processes, growth rates and reproductive cycles. However, we decided not to analyze this further because the lack of temporal evolution would limit the analysis for this parameter to the sampled settlements themselves. Temporal evolution within sites can be better visualized by simple box plots (S1 and S2 Figs). In addition, elevation can also influence precipitation patterns and thus soil moisture. The windward (luv) sides of mountains typically receive more precipitation than the leeward side, resulting in vegetation differences across north-south mountain ranges. Therefore, we can expect some co-determining relationships between RAP and elevation that may already be captured by RAP. In fact, the PCA we performed on the minimum Δ^13^C and elevation did not provide any additional patterns to those observed with RAP, as suggested by CCA.

Apart from RAP, another general geographic pattern can be observed when considering S1 Fig. Overall, fruit Δ^13^C values reflect the north-south and west-east decline in precipitation of the region, with the strongest stress in olive stones and grape pips indicated for Qatna (Syria), Tel Lachish (Israel) and Hirbet ez-Zeraqon (Jordan).

### Water availability for grape and olive and its relationship to reconstructed average rainfall

Although olive trees can survive with as little as 200–300 mm of annual rainfall, optimal growth and fruit production are better supported with 400–600 mm/a, up to 800 mm for high yields. The ideal annual rainfall range for today’s vines is typically between 600–800 mm/a, but total seasonal requirements vary between 500 and 1200 mm/a, depending mainly on climate and length of growing season (https://www.fao.org/land-water/databases-and-software/crop-information/en/).

Using hindcasted reconstructed average precipitation (RAP) as an environmental variable of the olive and grape seed and wood charcoal datasets, based on mean, minimum and maximum Δ^13^C values as functional traits, the PCA indicates that mean Δ^13^C have a stronger association with RAP than the minimum values of Δ^13^C (S3 Fig). This can be explained by the fact that RAP represents a random-based mean rainfall, in line with mean Δ^13^C data, which indicate moisture availability as an annual average for wood and a seasonal average for fruits, while minimum Δ^13^C indicate the strongest stress signal in both wood and fruits, which can be a result of different circumstances, including natural and human factors.

Since the mean Δ^13^C values reflect a more generalized picture of the growing conditions of the species, we also consider the minimum Δ^13^C to capture the maximum stress experienced by the plants at a particular site.

**Fig 4:**
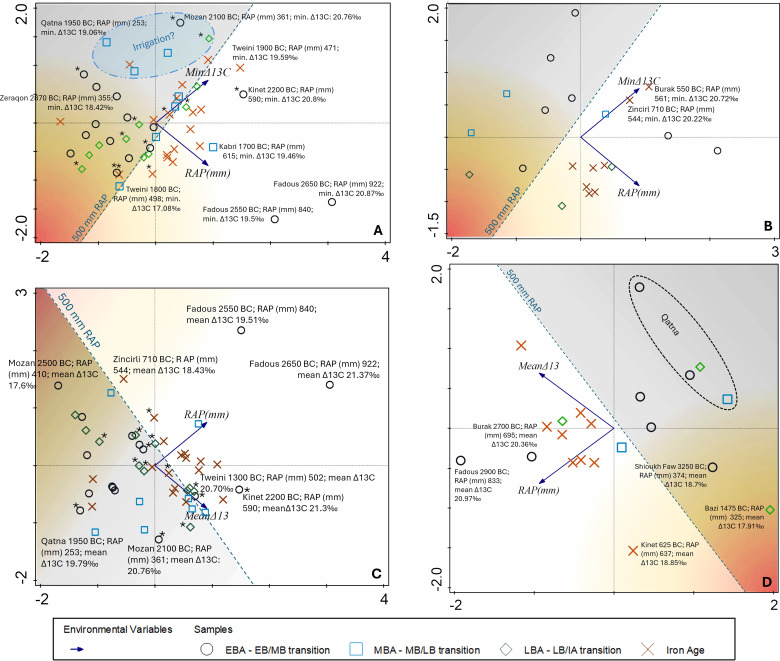
PCA of Δ^13^C values (means and minima) of grapes (wood and pips) and reconstructed average rainfall (RAP). (A) Minimum Δ^13^C values of grape pips, (B) Minimum Δ^13^C values of grape wood, (C) Mean Δ^13^C values of grape pips, (D) Mean Δ^13^C values of grape wood; asterisks next to the symbols indicate specimens that fall into the chronological ranges of the 4.2 and 3.2 ky BP events.

The PCA of four different data sets of Δ^13^C values (means and minima) of grapes (wood and pips) and RAP gives the highest explained variation with 77% for axis 1 with the mean Δ^13^C values of grape wood (Fig 4D). The most striking pattern is the accumulation of Iron Age samples from different sites well above the 500 mm RAP. For grapes, the explained variation of axis 1 is never less than 67%, supporting a high degree of association in the parameters Δ^13^C, RAP and chronological range of grape samples from Southwest Asian archaeological sites.

Considering the Δ^13^C minima in grape pips (Fig 4A), the associated principal components in the data set indicate that 47% of the variation is related to the close association of Δ^13^C minima with RAP, 33% is positively associated with the different chronological ranges, while 20% is negatively associated with the chronological ranges. In particular, these 20% of the negative association of Δ^13^C minima with chronology are of interest to explain locally unfavorable conditions for viticulture. Δ^13^C minima of Iron Age and Middle Bronze Age grapes are mostly plotted on the right part of axis 1, while Early and Late Bronze Age values are mostly plotted on the left part of axis 1 (Fig 4A). The minimum Δ^13^C values indicate a gradation of extreme stress signals with the higher values, but less extreme stress indicated in the upper right corner of the graph and the strongest stress expressed by smaller minimum Δ^13^C values in the lower left corner of the graph. Thus, the lowest overall stress signals come from the Middle Bronze Age samples, whether they are from sites below the 500 mm RAP, such as Qatna (253 mm RAP), or from sites above the 500 mm RAP, such as Tell Kabri (615 mm RAP). The 500 mm RAP can be considered the lower limit of non-irrigated viticulture. Higher yields might have been achieved well above this limit. Locations below this line were probably forced to irrigate the crop, either systematically or occasionally.

A similar pattern to the Middle Bronze Age can be observed for Iron Age grape seeds, although a significant number of these showing stronger stress signals. The majority of the Early and Late Bronze Age fruits experienced more extreme drought stress than the Middle Bronze Age and most of the Iron Age grape fruits. The minimum Δ^13^C values for grapes well below the 500 mm RAP (upper left corner above the 500 mm RAP line) come from Syrian sites (Qatna, Halaf) and from Zeraqon in Jordan (Fig 4A). All these sites are known for the presence of artificial or natural water reservoirs, which were probably also used for systematic or occasional irrigation. Sites slightly below the 500 mm line of the RAP include Lachish (RAP c. 400 mm) in southern Israel, but also sites in generally favorable rainfall regions. These date around the 4.2 kyr BP event (Tell Tweini 2200 BC, Tell Tayinat 2180 BC; marked with an asterisk in Fig 4A), and the 3.2 kyr BP event (Jaffa 1130 BC, Tell Tayinat 1200 BC, Tell Atchana 1200 BC). In short, these two climatic events are well reflected in the low RAP, and to some extent also in the Δ^13^C minimum values.

Although there are fewer wood samples for vines available, the PCA of the minimum Δ^13^C values and RAP (Fig 4B) show similar patterns to those observed for the grape seeds. Again, most of the Iron Age samples are above 500 mm RAP, i.e., cultivation during this period seems to have benefitted from increased water availability, which may in fact be a matter of the locations being placed in regions with higher rainfall. However, while most of the Early Bronze Age grape pip samples show more extreme drought stress in their Δ^13^C minima (Fig 4A), most of the wood samples show the opposite pattern, i.e., Early Bronze Age Δ^13^C minima that are less extreme than for the fruits, although in both cases most sites are located below the 500 mm RAP. These contrasting patterns can be best explained by fluctuations in seasonal water availability, with extremely low water availability in the summer leading to considerably increased stress during fruit formation compared to the annual water budget, which is reflected in the weaker stress signals in wood. The Δ^13^C minima of the few wood samples, on the other hand, indicate the highest stress for the Late Bronze Age and for most of the Iron Age wood samples, with the exception of Tell Burak and Zincirli, although most of the locations received more than 500 mm RAP (Fig 4B). This suggests that the annual maximum stress levels of the trees were lower in the Early Bronze Age than in later periods, despite most of the Early Bronze Age sites being located in regions with lower RAP. The seasonal maximum stress levels for fruits, on the other hand, were lowest in the Middle Bronze Age and partially also in the Iron Age, compared to the higher maximum stress levels for Early and Late Bronze Age fruit formation. Considering the differences in the maximum stress levels of the annual (wood) and seasonal (fruit) signals helps to clarify these trends and may even allow us to identify irrigation, especially for the sites with lower rainfall (Fig 10).

**Fig 5:**
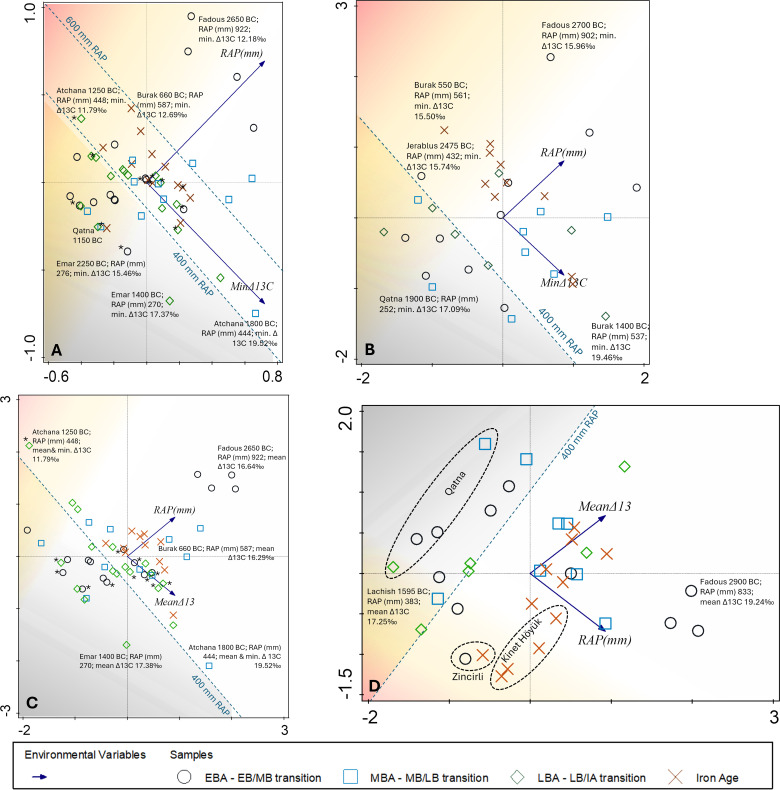
PCA of Δ^13^C values (means and minima) of olives (wood and stones) and reconstructed average rainfall (RAP). (A) Minimum Δ^13^C values of olive stones, (B) Minimum Δ^13^C values of olive wood, (C) Mean Δ^13^C values of olive stones, (D) Mean Δ^13^C values of olive wood; asterisks next to the symbols indicate specimens that fall into the chronological ranges of the 4.2 and 3.2 ky BP events.

**Fig 6:**
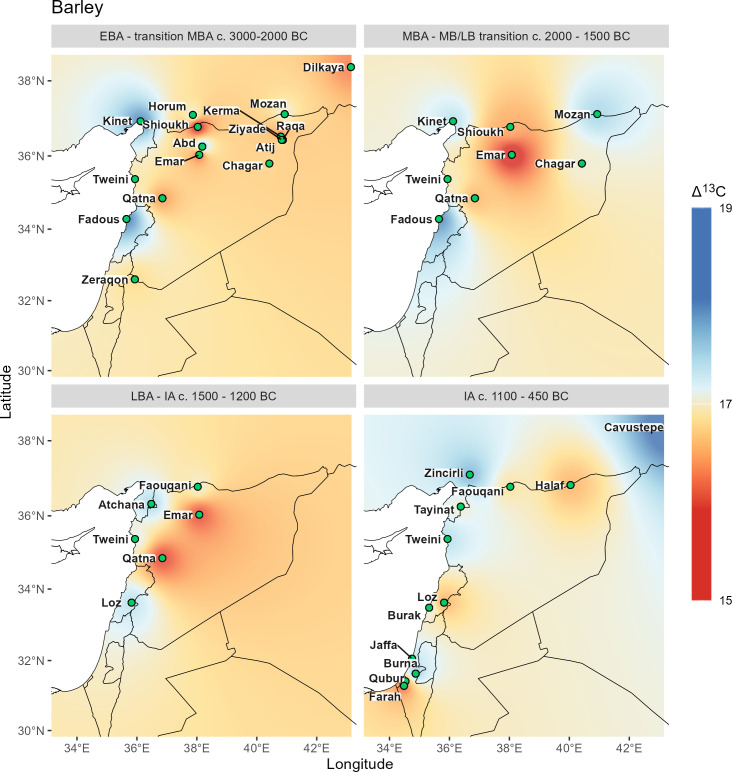
Δ^13^C interpolations for barley grains. Vector data provided by Natural Earth.

**Fig 7:**
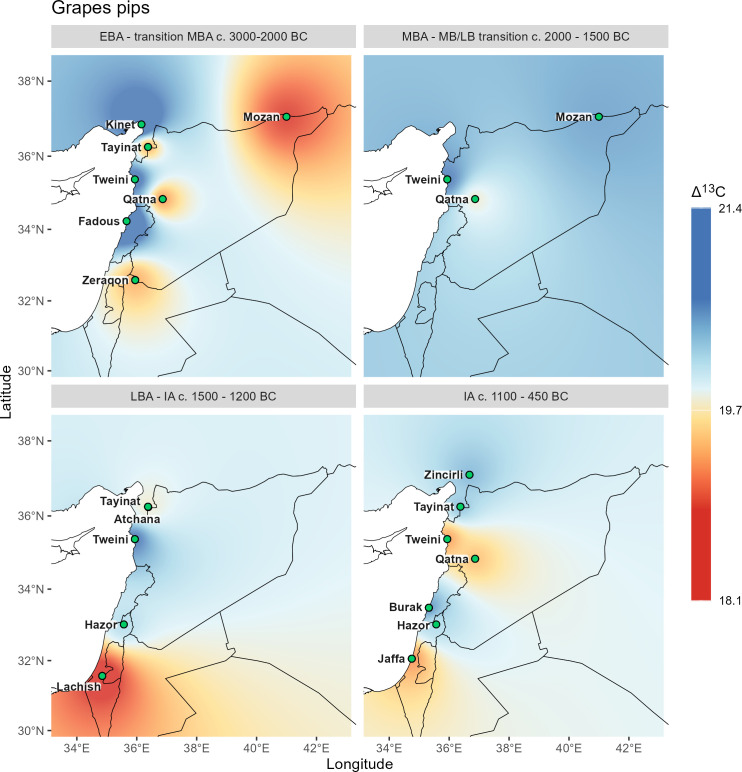
Δ^13^C interpolations for grape pips, based on stress levels suggested in this study (see ch. 3.1). Vector data provided by Natural Earth.

**Fig 8:**
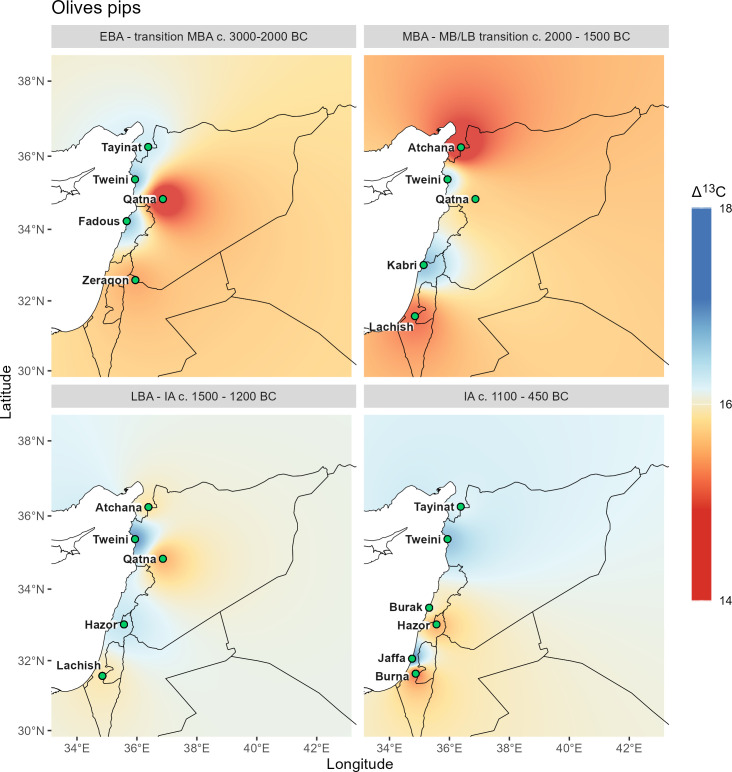
Δ^13^C interpolations for olive stones. Vector data provided by Natural Earth.

**Fig 9:**
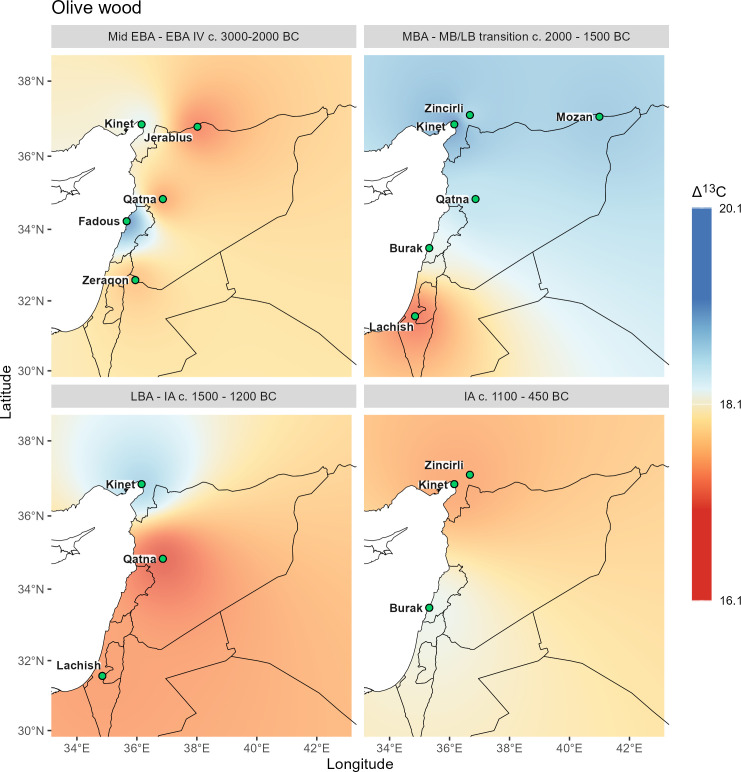
Δ^13^C interpolations for olive wood with color ramp based on thresholds outlined in ch. 3.1. Vector data provided by Natural Earth.

**Fig 10:**
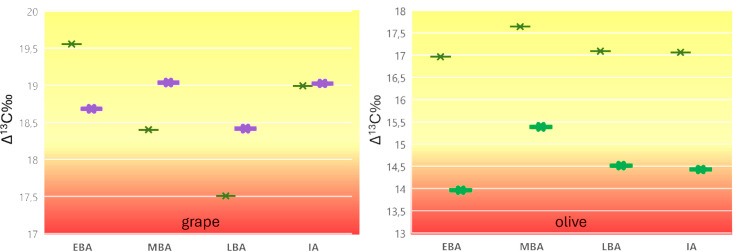
Maximum stress level for grape and olive over time as indicated by period means of the Δ^13^C minima. The lowest values indicate the most extreme drought stress (red), while the highest values indicate less extreme but still drought stress (yellow) for the specific time periods. The thick symbols refer to fruits, the thin symbols to trees.

The Δ^13^C means and minima of grape wood indicate that the Late Bronze Age samples and some of the Iron Age samples experienced greater drought stress than most of the Early Bronze Age samples. The mean Δ^13^C values in wood samples are all relatively high (Fig 4D), except for Late Bronze Age Tell Bazi, Early Bronze Age Tell Shioukh Tahtani in Syria, and Iron Age Kinet Höyük in the Hatay region of Türkiye. The site with the lowest RAP during all settlement phases is Qatna (Fig 4D). Nevertheless, the mean Δ^13^C values from Qatna are comparatively high, ranging between 19–20.5‰.

The minimum Δ^13^C values of the Iron Age pips are highly variable (Fig 4A); some show extreme stress for the summer months of their harvesting season, although they mostly come from sites within or above 500 mm RAP. This may be due to irregular irrigation or local differences in irrigation practices during a specifically dry summer season, which we could interpret as due to spatially variable agronomic principles and working practices.

Sites close to the 500 mm RAP include some settlements closer to the coast, such as Middle Bronze Age Tell Tweini, Late Bronze Age Jaffa, and also the Hatay sites of Tell Tayinat and Tell Atchana. It is particularly noticeable that most of the Middle Bronze Age and Iron Age vineyards received optimal amounts of water, as their seeds mainly show high mean Δ^13^C values. High mean Δ^13^C values of grape seeds in Middle Bronze Age settlements are represented by Tell Mozan, Qatna, Tell Tayinat, Tell Tweini, and Lachish. The fruits from these sites all have much lower mean Δ^13^C values during the Early Bronze Age and Late Bronze Age settlement phases. It is therefore likely that the Middle Bronze Age values reflect highly efficient irrigation during this period.

The PCA of four different data sets of Δ13C values (means and minima) of olives (wood and fruit stones) and RAP gives the highest explained variation with 74% for axis 1 with the mean Δ^13^C values of olive wood (Fig 5D), while the explained variation of axis 1 for the other data sets is lower, ranging between 68% for the mean Δ^13^C values of olive stones (Fig 5C) and 52% for the minimum Δ^13^C values of olive stones (Fig 5A). Nevertheless, all four PCA plots share a strong clustering of Iron Age sites above the 400 mm RAP, the lower limit of productive olive cultivation (https://www.fao.org/land-water/databases-and-software/crop-information/olive/en/). Assuming that our results are representative of the general conditions in the region, where moisture availability represents the annual average, it seems plausible that a shift of olive orchards to regions of generally higher moisture availability in the Iron Age increased agronomic productivity. Despite this, the minimum as well as the mean Δ^13^C values of olive wood suggest that a significant number of these Iron Age samples do not reflect ideal water availability (Fig 5B, D).

The Middle Bronze Age wood samples from settlements in the northern Levant and Syria, on the contrary, show comparatively high mean and minimum Δ^13^C values, indicating good water availability regardless of the range of RAP in which they are located, suggesting that the plants in these regions were well cared for during this period.

Since the minimum Δ^13^C values of olive stones provide the lowest explained variation of only 52% for axis 1, strong chronological associations are not indicated, except for the Iron Age cluster and a relatively large number of Early Bronze Age sites in regions of RAP lower than 400 mm (Fig 5A). The sites in the lower left quadrant of the plot that are located in regions below the 400 mm RAP, also include Late Bronze Age Qatna and Emar in inland Syria, Hirbet ez-Zeraqon in Jordan and Middle Bronze Age Lachish in southern Israel.

There are not enough wood samples from the critical chronological transitions to evaluate the effect of the 4.2 and 3.2 ka BP events on Δ^13^C but considering the pattern of mean Δ^13^C values in olive stones, the sites falling chronologically into these climate events all accumulate below and along the x-axis (Fig 5C), whereas the minimum Δ^13^C values for stones show no clear clustering (Fig 5A). The alignment of the samples along the x-axis indicates that they are associated with relatively high Δ^13^C means, which can be interpreted as a sign of special care and irrigation of the plants during these critical periods. Of the 11 settlements that fall chronologically into the 4.2 and 3.2 ka BP events, only Tell Atchana and Qatna produced a very low Δ^13^C value (Fig 5A, C). However, since only one olive pip from Tell Atchana was available for measurement, it is unclear where this value lies within the range of true means and minima. The majority of samples that fall chronologically into the 4.2 and 3.2 ka BP events and have relatively high mean Δ^13^C values are in descending order Jaffa and Tweini at the end of the Late Bronze Age, Tweini and Tayinat at the end of the Early Bronze Age, Hazor, Atchana, and Lachish at the end of the Late Bronze Age, and Emar at the end of the Early Bronze Age. Most of these sites are also located in regions above the reconstructed 400 mm RAP, but it can be assumed that the farmers at the few settlements below the 400 mm RAP level probably irrigated their crops.

### Interpolation

All Δ^13^C interpolations reflect the general north-south and west-east gradient of precipitation, which varies for individual species and also shows local differences of water availability in a diachronic comparison.

Δ^13^C values of barley from Near Eastern archaeological sites have been extensively studied and discussed previously [28,74,75]. As a drought-tolerant cereal with a short life cycle, it has been cultivated mostly under rain-fed conditions and thus primarily reflects the natural water availability at the growing sites [30,76]. For barley we see relatively similar patterns throughout the Bronze Age, with slightly increasing drought signals inland and in the north. Unfortunately, there are no Bronze Age barley data for Israel, so the dynamics in the southern Levant for this crop remain undetermined. For the Iron Age, drought stress signals are generally less extreme, which has already been visible in the PCA patterns, and relatively good moisture conditions are evident along the Levantine coast as far south as Jaffa.

Comparing the barley grains with the grape pip interpolations (Figs 6 and 7), distinct differences can be described, which can be partially explained by differences in water management at different geographical locations.

The Early Bronze Age patterns are relatively similar for both crops, but the stress signal of grapes at Tell Mozan are comparatively higher than that of barley, suggesting that water stress for grape is related to comparatively high aridity at the site despite possible irrigation (Fig 4A). In contrast, the stress signal for grape at Tell Tweini is significantly lower than for barley, indicating locally high water availability for grape, which could be due to direct irrigation of grape or to different cultivation areas selected by the farmers according to the different water requirements of the two species.

For the Middle Bronze Age only three sites are available for grape. For Tell Tweini the pattern remains the same as in the Early Bronze Age and continues to demonstrate much better management of growing conditions for grape than for barley. The pattern for barley suggests a considerable increase in aridity at the Euphrates site of Emar from the Early to Middle Bronze Age, while for the Upper Khabur region (Tell Mozan and Chagar Bazar) moisture availability for barley as well as for grape is much improved during the Middle Bronze Age. The coastal conditions change little with only a slight decrease in moisture availability for barley at Kinet Höyük. At Qatna, stress levels for barley are slightly lower in the Middle Bronze Age and conditions for vine cultivation are considerably better, suggesting that the vineyards were irrigated during the Middle Bronze Age.

While there are no Late Bronze Age data for barley from sites in Israel, the patterns in the north and interior suggest relatively similar conditions to those of the Middle Bronze Age. These are complemented by grape data from Tel Lachish, which indicate highly stressed Late Bronze Age vineyards in the south, while those at Hazor were better off. Grapes seem to have continued to be irrigated at Tell Tweini, while at Tell Atchana some drought stress for grapes can be noted.

The Iron Age growing conditions for barley appear improved with generally lower drought stress signals. However, vineyards seem to have experienced some drought stress at many sites, in particular at Tell Tweini.

Olive stone interpolations require comparison with olive wood interpolations (Figs 8 and 9), as well as those for grape seeds and barley (Figs 6 and 7).

Despite different locations in the north, the broadly annual signals for olive wood and the seasonal signal for its stones are almost identical in the Early Bronze Age interpolations, except a significantly higher seasonal stress signal of the olive fruits at Qatna. The general pattern for olive follows the average rainfall, with wetter conditions along the coast. However, the most striking pattern when comparing the four sample categories (olive wood, olive pits, grapevine seeds, and barley caryopses) is the near congruence of barley and olive signals, even though considerably more localities were sampled for barley. This suggests a similar treatment of both crops with regard to water availability during the Early Bronze Age.

The congruence of water availability for the plants and the general layout of isohyets is also visible for grape, although the early winegrowers paid special attention to the crop, either by cultivating specifically in locations close to water or by irrigating the grapevines in contrast to the other crops.

For the Middle Bronze Age, the olive wood shows distinct differences from the Early Bronze Age patterns and also from the olive stones. The wood interpolation shows an extreme north-south decline in moisture availability, but no differences between coastal and inland regions as in other maps, despite sufficient sites. It is particularly striking that olive wood from the Syrian sites of Tell Mozan and Qatna indicate high water availability, even higher than at the Lebanese coastal site of Tell el-Burak. Only the olive trees from Tel Lachish in Israel show drought stress which is in line with the olive stones from this site. The annual patterns contrast particularly with the seasonal drought stress of fruits from Tell Atchana in the Hatay region, and also from Qatna, while for the south the patterns are consistent with fruits. Since the minimum Δ^13^C values of wood from the northern sites are also very high (SI stable isotope data.xlsx), the pattern is difficult to explain but could indicate particularly high winter precipitation followed by extremely dry spring and summer seasons. In the case of Tell Mozan it is possible that the wood was imported as no olive stones have been found at the site so far notwithstanding intensive research took place [11,48]. Even if the Middle Bronze Age interpolation of the Δ^13^C values for grapevine seeds are not representative with only three localities, they nevertheless underline the good water availability indicated for the olive wood in the northern part of the study area. However, as no measured values for grape pips are available for the southern part, no statement can be made on the possible congruence of the two maps. The values for barley, with the additional data point of the Euphrates site Emar, offer a better differentiation of the regional patterns and indicate that both the northern Levant and the upper Khabur area had good water availability during the Middle Bronze Age. A superposition of all four patterns would give a similar picture as for barley, with high stress signals in the middle Euphrates area and in the southernmost part of the southern Levant and more favorable conditions in the upper Khabur area and in the central part of the coastal Levant, while the Hatay area still produces contradictory signals with the very high stress signal of the olive pits from Tell Atchana. An alternative hypothesis, requiring further stable isotope measurements, including strontium, is that olive fruits at some sites arrived from completely different growing areas, perhaps through long-distance trade.

The Late Bronze Age patterns for olive wood contrast with those of the Middle Bronze Age in that they show high drought stress for Qatna, whereas the Δ^13^C values for fruit show consistently low or no water stress, with in general much lower values than in the Middle Bronze Age. This suggests that olive orchards were generally irrigated during the fruiting season in the Late Bronze Age. Interpolations for grapevine seeds are limited by a small number of localities for the Middle Bronze Age, but the Late Bronze Age patterns indicate drought stress for the southern part of the southern Levant, whereas the vines in the central and northern part of the Levant hardly experienced any water shortage. The patterns for barley are very similar to those of the Middle Bronze Age, but there are no measurements for the upper Khabur area.

The most striking pattern in the comparison of the Δ^13^C values of the Iron Age with earlier periods is the absence of extreme stress signals in all cultivated plants. This also applies to olive wood, although its representativeness is limited to only three localities. The earlier alignment of Δ^13^C values with the general layout of isohyets has been replaced in the Iron Age sites by local variability, which is particularly visible for grape, indicating moderate stress at coastal sites. The interpolations for barley and olive stones are very similar, as has already been noted for the Early Bronze Age.

## Discussion

### Interpretative insights from traditional grape and olive cultivation in the Levant

Information on traditional farming methods can help interpreting isotope signals, as ancient techniques may have altered physiological processes in plants in ways that have no equivalent today.

Traditional land use patterns in the northern and southern Levant in the 20^th^ century are dominated by cereal and vegetable cultivation and oleiculture, with vineyards occupying only a small proportion of arable land and the majority of grape and olive orchards not being irrigated [77,78]. In the context of this broad cultivation tradition, we can compile further details from older written sources and ethnographic literature to ascertain whether they have any influence on the physiology of the plants. This will facilitate a more profound understanding of the deviations from the general pattern that arise from meta data analysis.

### Seasonal conditions during fruit formation

In accordance with the natural life cycle of crops, agricultural work is carried out in a regular sequence. For example, barley typically takes 2–3 months to fill, grapes 3.5–5 months from flowering to harvest, and olives 5–7 months. This results in a traditional harvest sequence for the Levant of grain followed by grapes, both of which must be completed before the rainy season, because the onset of rain or heavy dew makes it impossible to leave grain and grapes out in the open [79]. Olives are harvested afterwards.

Depending on the timing of flowering and fruiting, this results in different water balances during the ripening of the different crops. Irrespective of the possibility that irrigation may have played a role in areas with low mean annual precipitation, the Δ^13^C values of the three different crops generally reflect the mean moisture availability of different seasons, which, according to the ethnographically reported harvest calendar, would be the extended season from roughly May to October for olives, with the harvest starting no later than mid-September [80], while for grapes it would be the somewhat shorter period between May and July, depending on the geographical region, and for barley a very short sequence in late spring [79].

According to the annual rainfall pattern in the cultivable lands of the Levant, the months from June to September are more or less free of precipitation. Due to the shorter ripening period of the fruits of grape, the plant is exposed to a relatively shorter dry season than the olive during this physiologically demanding period but may be extended in some cases when raisins are produced [80]. The predominant growth of olive fruit during a prolonged dry season thus explains, at least in part, the relatively low Δ13C values compared to grapes and also compared to olive wood, which preserves an approximate annual precipitation signature rather than a seasonal signal. Olive wood stores carbon assimilated under favourable conditions, that can be remobilized upon stress conditions when photosynthesis is limited [81], while olive fruits depend more on freshly assimilated carbon [66]. The relatively small difference between the Δ^13^C values of grape wood and pips, on the other hand, might support the argument that the grape was generally better watered or likely irrigated, probably also much earlier in history than olive (Fig 2).

### Water use efficiency and irrigation

Biblical sources also provide comprehensive insights into water management practices in olive orchards and vineyards. Olive trees are considered to be efficient water users on rocky soils because their roots penetrate into the crevices of the rock to take advantage of the moisture content of the deeper layers which also enables them to thrive on south-facing mountain slopes. Olive trees are often placed on land that is too stony to support cereal crops. Ethnographic sources also show that olive and vine are usually not grown together because of the very different care they require [80]. In order to minimize the competition for nutrients and water between the plants, the olives are planted in rows about 10 meters apart and within the rows about 5 meters apart, but over time the original order is often broken. The olive groves are plowed or hoed three times a year, depending on the type of soil (at the beginning of the rainy season or in February, and again in April and May). The first plowing is to open the soil for the winter rains, the second and third to remove the weeds that have grown during the rainy season. Occasionally, a small pile of stones is placed around the trunk to prevent evaporation of soil moisture. In addition, a pit is often dug around each tree at the end of January so that rainwater can collect and penetrate deeper. In the southern Levant, artificial irrigation is usually impossible because the olive groves are rarely located below the springs. This is also supported by the Hebrew Bible, which refers to most of the orchards being in mountainous regions and thus not being irrigated [82].

Since the southernmost limit of the natural distribution of wild *Vitis vinifera sylvestris* in the Levant is at the northern edge of the Sea of Galilee [83], which is also supported by ethnographic records [80], and along a narrow strip extending from the Hatay region eastward along the Turkish-Syrian border [13], it can be expected that most of the samples we examined from the southern Levant and from northern Mesopotamia were derived from cultivated plants that required special care to compensate for natural limitations in their physiological requirements.

Traditional as well as biblical records list numerous techniques of protection and treatment to promote healthy development of the fruit, including the construction of guard huts in the vineyards to keep animal predators away [80]. The Hebrew Bible specifically mentions the importance of soils, which should be rich and free of rocks, which corresponds to the major regions of grape cultivation in 1880 [78], and with Terra Rossa as documented in the soil map of Israel [84]. Depending on the region, the grapes are traditionally harvested in July/August, with the main harvest in September, which can extend into November in mountainous areas. However, such a late harvest is only possible if the grapes are shaded with linen cloth, a practice described for both Israel and Lebanon [80]. Traditional economic measures generally include delaying the ripening process by covering the vines to sell the thereby artificially lowered stocks of grapes at a higher price. This represents a significant manipulation of the vines in terms of water use efficiency and photosynthesis, thereby affecting the Δ^13^C signals.

Overall, grape cultivation is much more labor-intensive than olive cultivation in terms of soil preparation, protection from predators, and irrigation during the fruiting season. These features and its sensitivity during the flowering season are well expressed in the saying *eddālie sirīja ez-zētūn bedawīja...* translated as “The vine is a lady, the olive is a Bedouin,...” [80]. This saying reflects the survivability of olive from the perspective of the traditional farmer in the southern Levant. The biblical texts also attest to the considerable cultural and symbolic importance of vineyards, including their importance for the higher social status of their owners [82].

Although irrigation is considered beneficial for increasing productivity, Dalman [85] questions its necessity for olives and grape, but in mentioning that olive plantations in Tafilah southeast of the Dead Sea and grape plantations in Damascus are irrigated, he acknowledges the purpose of increasing yields. Biblical texts for grape (Ezekiel 17:5 ff.) and other fruit trees (Psalm 1:3, Jeremiah 17:8, Ezekiel 47:12) indicate that irrigation is considered beneficial, though not essential. However, biblical texts explicitly refer to irrigated crops in order to emphasize the general prosperity of the landscape (Dalman [85] and [80]).

Dalman [85] also notes that in the vineyards, the trunks of the grapes lay on the ground and the vines grew like bushes, their leaves covering the ground and protecting the grapes. This type of cultivation may have protected the grapes from wilting during periods of drought. He also describes grapevines growing over a tree. Wedded grapes were documented in the Hittite texts [86] and in iconographic sources from the Neo-Assyrian period [87]. Dalman [88] reports that long vegetable patches were sometimes planted between olive groves. In this case the olives also benefited from the occasional irrigation of the vegetables. The particular cultivation techniques also influenced the soil conditions.

As can be seen from the biblical sources, irrigation and especially manuring of vineyards did occur, but it was not considered the norm, perhaps because the purity of the fruit seemed to be at risk. Such aspects are a matter of fine-tuning and can only be assessed by further implementation of other stable isotope measurements (e.g., nitrogen and strontium) or with a deeper insight into the local environmental and cultural developments. However, it should be borne in mind that they may have varied at different locations, thus leading to the regional differences documented in our Δ^13^C values.

### Contextualizing fluctuations in the archaeobotanical and stable isotope record

Diversity in local and species ecology and their interrelationship with human actions complicate the interpretation of stable isotopes in plant remains. The following sections are intended to critically question linear interpretations of Δ^13^C and growing conditions.

### Ecological background patterns of grape and olive finds

Biomolecular studies of olives indicate at least eleven ancestral populations in the Mediterranean, with at least nine domestication events [89–91]. Half of the cultivars result from crosses of single or multiple lineages distributed in both the western and eastern parts of the Mediterranean region. Genetic studies provide evidence of the olive’s wild ancestors in the Levant region and underpin the olive tree’s evolutionary resilience to the ecological conditions of the Levant and the species’ general drought resistance [92,93]. The cultivation areas of olives are clearly limited to the Mediterranean region due to their sensitivity to frost. Their fruit development requires average temperatures of 18–22°C, but they can tolerate temperatures up to 40°C. Seasonal cold snaps with temperatures around freezing point have a detrimental effect, so that the plant generally does not survive prolonged periods of frost [89]. The generally earlier mid-Holocene pollen record in the southern Mediterranean compared to more northern regions [90] may be related to the temperature drop associated with orography. The Lebanon, Anti-Lebanon, and Taurus mountains limit the possible habitats for olive cultivation to very narrow strips along the coastlines with elevations below 800 m, which even decrease towards the north [91].

The current distribution area of the wild grapevine in Europe and the Middle East includes not only the circum-Mediterranean region but also the northern Fertile Crescent, the entire Black Sea coast and areas east of it as far as the Caspian Sea, which is in line with the palaeogenetic evidence of multiple centers of domestication and diversification with introgression also playing a role [9,94]. The fact that the grapevine is more widespread than the wild olive is largely due to its greater habitat flexibility. Wild grape can be found in a wide range of habitats, including coastal areas and beaches, forests and riverbeds. It should be emphasized that habitats at altitudes below 300 m with access to water sources, high vegetation density, low anthropogenic disturbance and potential correlation with carbonate soil substrate are generally more favorable for the establishment of stable populations of wild grape [83]. For domesticated vines, the northern limit of cultivation is slightly higher than the 50th parallel, provided that the growing season includes at least 180 frost-free days with mean daily temperatures not falling below 15°C as a long-term average [95].

The ecological differences affecting the natural distribution of olive and grape are also reflected in the archaeobotanical data, with a relatively narrow distribution of olive in the Levant and the extension of grape finds beyond this area into northern Mesopotamia [11,96], (Fig 1).

A morphometric study of Chalcolithic olive finds from Hishuley Carmel in Israel revealed a very high diversity of forms, some of which resemble modern cultivars [97]. Studies on grape pips dating to the Early Bronze Age until the Hellenistic period suggest similarly large numbers of varieties for the upper Euphrates region [98]. Since modern crop varieties and cultivars have different agronomic requirements, it should be taken into account that archaeobotanical specimens that differ greatly in their morphometric measurements, often referred to as different phenotypes, may also have had quite nuanced ecological properties. This could also have led to minimally different responses to water stress, which in turn may have contributed to the complexity of the Δ^13^C measurements.

### Water availability: Can we disentangle climate and irrigation in the stable isotope record?

Comparing the Δ^13^C values of wood and fruit of tree species and considering their general requirements for water availability in relation to reconstructed rainfall, it is theoretically possible to distinguish between rainfed and irrigated cultivation. There is a tendency for a greater difference in Δ^13^C values of wood and fruit at more arid sites compared to sites in regions with higher rainfall (e.g., compare values for olives from inland Syrian Qatna with those from coastal Lebanese Tell Fadous in S2 Fig). Apart from the greater likelihood of irrigation in low rainfall areas, we can use the difference between the annual signal in wood and the seasonal signal in fruit to distinguish irrigated from non-irrigated fruit. If the difference between the wood and fruit signals at a site in an arid region, such as Qatna, is very small or even non-existent, it can be assumed that the fruit was irrigated. With the exception of the olive values from Late Bronze Age Qatna and Lachish in Israel, this pattern is not evident for the other sites where we have both charcoal and fruit stones (S2 Fig). At sites with higher rainfall, such as the Lebanese sites of Tell el-Burak around 450 BC and Early Bronze Age Tell Fadous, there can be even an overlap of the Δ^13^C values of wood and fruit.

For vine, there are far fewer Δ^13^C values for charcoal available for comparison with those for seeds, but here, on the basis of the lack of difference in the Δ^13^C values in charcoal and seeds, irrigation of the vines for Qatna around 1900 BC and perhaps also for Tell el-Burak around 660 and 450 BC must be a possibility, despite the comparatively high rainfall at the latter site. This is in line with a general pattern in the later Territorial Empires of northern Mesopotamia to irrigate in areas even where there is high rainfall. Wilkinson [99] relate this to the desire to maintain stable yields, rather than raise yield, for tax reasons.

### Grape

PCA plots showed an association of Iron Age and Middle Bronze Age Δ^13^C minima of pips and separately of Early and Late Bronze Age values (Fig 4A). These associations could be related to either natural or human factors and require further discussion. Since these relationships are much weaker in the PCA of the mean Δ^13^C values, they might be related to the specific local circumstances of the maximum stress levels rather than to general mean conditions of viticulture. The PCA of minimum Δ^13^C of grape pips and wood samples also showed some contrasting chronological patterns, suggesting differences in seasonal water availability beyond the natural precipitation cycle. This was particularly evident in the Early Bronze Age samples, which showed comparatively lower stress levels in the wood than in the fruit samples. A simpler and summarizing form of visualization of the different data sets makes the overall patterns of contrasting water status of the plants clearer, and is applied here to consider the maximum stress levels of grape and olive (Fig 10).

For this generalization, the maximum stress levels in grape and olive were calculated on the basis of the minimum Δ^13^C values of the wood and fruit samples over time, since the minimum Δ^13^C values represent the maximum drought stress experienced by the plant tissue during the period of its formation. To obtain a representative minimum value per plant part and temporal range of all sites, the means of these minimum values were plotted for each period and species. The values thus indicate the differences in maximum stress experienced annually (wood) and seasonally (fruit) (Fig 10).

Compared to olive, the chronological development of seasonal and annual stress levels for grape is inconsistent, supporting the different trends in fruit and wood described for the PCA patterns. Grape wood shows a continuous increase towards more extreme stress levels from the Early Bronze Age to the Late Bronze Age, best interpreted with the mid-Holocene drying trend observed in many palaeoclimate proxy records [100], while the less extreme annual stress level of grape in the Iron Age is consistent with the PCA patterns. Compared to olive stones, the values for grape fruits are less extreme, with a low inner-site variability throughout time (S1 Fig). This supports stronger agricultural control of the vines than of olive trees through intense management and irrigation, especially at the Middle Bronze Age sites (mean minimum Δ^13^C for fruit is > 19‰), to some extent also at the Late Bronze Age sites, and to a lesser extent at the Iron Age vineyards, which were in regions of generally higher rainfall (RAP). The contrasting more extreme seasonal stress signals of the Early Bronze Age fruits compared to the wood suggest that the vines were probably not irrigated during the summer in the Early Bronze Age and thus suffered more from summer drought than in later periods.

Using the maximum stress of wood and fruits allows distinguishing between natural moisture availability and irrigation to some extent, but other factors such as cultivation location and the possibility that the fruit stones found in a site derive not from the same trees where the wood has been taken from must be also considered to evaluate this question on a case-by-case basis. For example, grape irrigation can be assumed for samples with relatively high minimum Δ^13^C values from settlements well below the 500 mm RAP (marked with “irrigation?” in Fig 4A), such as Qatna, Tell Mozan, and Tell Halaf. These settlements are located in present-day Syria and were probably prone to interannual rainfall variability, so their inhabitants may have applied irrigation to grapevines. This is also well reflected in the PCA of the mean Δ^13^C of the wood samples, with Qatna producing comparatively high values despite its location far below the 500 mm RAP threshold (Fig 4D), indicating that the vines were probably irrigated using the huge water reservoirs at the site (cf. [101]. The settlements close to the 500 mm RAP could also have been affected by these natural yearly fluctuations, and in particular during the climatically induced dry periods, such as the 4.2 and 3.2 BP events, irrigation can be concluded to have been applied to avoid yield losses.

### Olive

The PCA for olive, similar to that for vines, suggests that Iron Age cultivators followed a different strategy than their Bronze Age predecessors, profiting from higher rainfall. Nevertheless, some of the selected sites were not optimally suited to the needs of the plants, so that some of the specimens experienced drought stress despite being located in a relatively favorable location. The Middle Bronze Age specimens contrast these patterns in that they were distributed over a much wider range of RAP, but nevertheless generally had higher mean Δ^13^C values, which was particularly evident in the wood samples. That the plants were well cared for during this period is also evident in their low maximum stress values (Fig 10), which show the least extreme stress for trees and especially for fruits during the Middle Bronze Age.

The highest drought stress, plotted in the PCA of the lowest minimum Δ^13^C values of olive stones in the upper left corner of the diagram (Fig 5A), is evident from the settlements of different periods and rainfall ranges. Very high Δ^13^C minima, on the other hand, indicating less extreme drought stress, are likely to represent irrigated fruits.

Specifically in relation to the 4.2 and 3.2 BP events, irrigation could have been considered even in regions that were generally well suited for olive production. An example is provided by the mean Δ^13^C values in olive stones (Fig 5C). Many of the sites that fall chronologically in the two major climatic events are above the 400 mm RAP and most of them show high mean values, indicating sufficient water availability. High mean Δ^13^C values in olive stones from regions below the RAP, such as the Syrian inland sites of Emar and Qatna, as well as Lachish in the Shephelah in the southern Levant, indicate that cultivation was at least occasionally carried out with irrigation during the fruiting season.

A recent study of Δ^13^C in modern and ancient olive stones in the southern Levant addresses ancient climatic fluctuations [61]. The authors define a Δ^13^C threshold for olive fruits under severe drought stress for values below 15.5 ± 0.5‰ by relating δ^13^C values of modern cellulose of olive stones to an aridity index. Since no significant difference was observed between fresh and charred modern olive stones at 250°C, the Δ^13^C values for modern stones can be directly compared to ancient material. A major advantage of this study is that the modern samples allow a clear distinction between irrigated and non-irrigated fruit. Notably, values above 16.5‰ were measured in irrigated plants. Applying this value to the means of our stable carbon isotope data, it might be argued that olives derived from irrigated trees at Jaffa (c. 1130 BC), Tel Kabri (c. 1800 and 1700 BC), Hazor (1200 BC), Tell Burak (c. 600 BC), Tell Fadous-Kfarabida, Tell Tweini in all periods except 1600 BC, Kinet Höyük (c. 1340 BC), and Tell Tayinat (c. 2180 BC and 980 BC). However, RAP for our sites of investigation have to considered as well. In the study by Ehrlich [61] modern irrigated crops in arid regions such as the Southern District can still experience high stress (about 12.5‰), as can unirrigated crops growing under comparatively adequate natural moisture availability (annual precipitation of about 360–560 mm in the study by [61]). Some of our samples mentioned for mean Δ^13^C above 16.5‰ coincide with the timing of the 4.2 ka BP and 3.2 ka BP events, and this may provide some additional support for irrigation practices in olive plantations at these times.

If we apply the threshold of below 15.5 ± 0.5‰ suggested by [61] as indicative of severe drought stress to our archaeological data, considering only sites and phases that produced at least three individual measurements, a number of sites and phases fall with their means slightly below this threshold. These are Lachish (c. 1470 BC), Tel Burna (c. 780 BC), Hirbet ez-Zeraqon (c. 3070 BC and 2870 BC), Qatna (c. 2500 BC, 2100 BC, 2000 BC, and 1400 BC), Tell Tweini (c. 1600 BC), Tell Atchana (c. 1500 BC). Many of these fall in the transition from the Middle to the Late Bronze Age, while Holocene climatic fluctuations in Southwest Asian history have been discussed mainly for the Early to Middle Bronze Age transition (4.2 ka BP event) and the end of the Late Bronze Age (3.2 ka BP event). Only a few publications have also mentioned aridity peaks towards the end of the Middle Bronze Age [102,103].

Apart from locally deviating patterns, the overall differences between trees and fruits, as seen in the maximum stress levels, can be described as the general background. The most striking pattern is the largest difference between fruits and trees of olive and grape during the Early Bronze Age (Fig 10). For both crop species, the stress signals in fruits are much stronger than in their tree counterparts than in any of the following periods, suggesting the most seasonally different growing conditions during the Early Bronze Age compared to later periods. This suggests that Early Bronze Age olive growers were more dependent on the natural water balance, whereas later they were more reliant on additional irrigation. In the Iron Age at latest all locations fall into the range of above 400 mm RAP.

In the case of grapevine, the relationship between wood and fruit values is even reversed from the Middle Bronze Age onwards, with lower stress levels in fruit compared to wood, which may indicate year-round additional water supplies for grapevines. In the case of olive, the differences in stress between fruit and wood became only slightly less pronounced from the Middle Bronze Age onwards. This supports an interpretation of a general evolution of cultivation, from none to minimal irrigation of the fruits in the Early Bronze Age, to more regular irrigation of grape and occasional irrigation of olive from the Middle Bronze Age onwards.

### Natural and man-made niches in the cultivation of fruit trees and the development of production centers

The well-studied RCC (Rapid Climate Change) events at 5.2, 4.2 and 3.2 kys BP (e.g., [104–109], etc.), have been argued to have a variety of impacts on ancient agriculture [30,110–112], etc.). Despite recognizable environmental effects on food production, attempts to generalize these over larger spatial and temporal units have so far proved to be inconclusive [28,113,114]. In particular, the role of RCC events on human societies, while potentially devastating to short-term production capacity, is regionally inconsistent. It has been suggested that population became increasingly decoupled from climate variability over the course of the mid-late Holocene, despite, or perhaps because of, increases in absolute numbers of people [100,115], which indirectly supposes accompanying expansion of production levels and cultivated areas. Population growth and the associated expansion of settlements, with a corresponding increase in demand for these goods, can also increase production levels [11]. Furthermore, in relation with expanding cultivation areas, the investigation of risk buffering strategies requires more attention [116].

While focusing on the primary environmental and human-technological factors to explain crop patterns, other causes likely contributed to changes in agricultural production, such as political instability, conflict, and war, which can lead to a lack of care due to a lack of labor, or even the destruction of vineyards and olive groves, as in the case of Ugarit [117]. However, scholars urge that the collapse and destruction of settlements that are readily attributed to natural disasters or wartime invasions require local reassessment [118].

The current evidence suggests that differences in regional and local climatic impacts and human actions to ensure sustainable yields require adjustment of generalized models with additional proxies, for example by accounting for agricultural niche construction processes at specific sites.

Database queries show differences in find quantities [11], although it is also differences in archaeobotanical sampling strategies that contribute to such patterns. In the Early Bronze Age Levant there is a conspicuously high proportion of olives from the Jezreel Valley (Megiddo), the Shefela (Tel Yarmuk), and southwest of the Dead Sea (Arad), which stretches into the southern and central highlands (Shilo and Tel Manahat) during the Middle Bronze Age, and shifts to the northern coast (Tyre, Sidon and Tell el-Burak) in the Late Bronze Age [119]. This northward shift might be related to the general Holocene increase in aridity. There might be some support from Δ^13^C values in olive wood (Fig 9), but more sampling locations are required to test this hypothesis. Especially in the Iron Age II, olive is documented in numerous sites, sometimes with very high proportions. A closer look at the predominant find contexts in the southern Levant shows that olive stones are frequently found in public buildings in the Early Bronze Age (e.g., Bet-Shean [120] or Ḫirbet ez-Zeraqon [37,38]). In the Middle Bronze Age, the diversity of find contexts is much higher, despite fewer sites [121–125]. In the Late Bronze Age, very large quantities of olives were frequently found at coastal sites, which might be related to the increasing surplus production for maritime trade [40,126,127]. The number of archaeobotanically investigated sites increased, especially in Iron Age II with a further increase of olive percentages.

Certain regions stand out over time with particularly high proportions of grape seeds. These are the coastal plains of Sidon and Tyre and further south and the Shefela in the east, the middle Jordan Valley, the central and southern highlands, and the southern eastern shore of the Dead Sea. With the exception of the latter area, all the sites are located in regions that now receive an average annual rainfall of about 400–700 mm or are on the middle reaches of the Jordan River and can therefore be considered favorable for grape cultivation. As can be seen from the Δ^13^C records in grapes, irrigation must have played a role in some settlements, especially in those located in regions of low RAP, such as the Syrian sites and probably also some of the sites in the southern Levant, e.g., settlements located in the Jordan River valley, while for the Iron Age we can assume that intensive viticulture in general was mostly practiced in regions of higher rainfall and regularly irrigated.

With reservations regarding the different sampling and sample processing strategies, it is noticeable that higher proportions of grape seeds are found in the southern Levant during the Early Bronze Age, especially in the Middle Jordan Valley, and from there in the following periods increasingly in regions closer to the coast. Strikingly high numbers of grape seeds have been recovered from Late Bronze Age contexts which are sometimes unrelated to wine production, such as probable remains of animal feed at Tel Aphek [128] or the raisins from Ashdod [129]. From the Iron Age onward, grape pips appear in greater numbers throughout the coastal region, which is probably related primarily to higher precipitation levels compared to inland locations, as supported by the interpolation patterns (Fig 7), but possibly also due to maritime trade networks. As noted in the Δ^13^C minima values of grape pips, there are indications of higher drought stress signals in the Early and Late Bronze Age samples compared to the Middle Bronze Age and Iron Age (compare also the strength of the drought stress signals in grape interpolations, Fig 7), but these only occasionally coincide chronologically with the timing of the 4.2 and 3.2 BP events (Fig 4A). The higher drought stress signals in the Early Bronze Age may be due to irrigation techniques that were not yet fully established in some regions and thereby causing irregular water availability for the plants, while the shift to the coast in the Late Bronze Age could have been a consequence of increasing surplus production and maritime trade networks despite comparatively high drought stress levels also during this period.

Based on these considerations, we may conclude that our Δ^13^C data reflect cultural niche-construction processes in agricultural systems in the sense of Dawkins, Laland and Odling-Smee [130–132]. For example, the decision to grow crops in a different location than before will not only change the landscape but will also change the composition of the field flora and the nutrient and water status of the crops, which will affect yields and the environment, and thus further influence human decisions. However, it must be kept in mind that humans are not the only modifying agents, and other organisms or natural forces may influence the niche construction process in varying degrees and frequencies, thereby inducing further co-evolutionary adaptation and niche construction. The cultural response to each change carries additional connotations of knowledge, traditions, and perceptions that are intertwined with biological and ecological change. The possible parameters of niche construction are manifold, and a single niche construction activity can simultaneously represent a cultural response, creating a continuous evolution. Because agricultural niche construction varies in space and time, the study of agricultural niches helps to better understand the relationships within and between different societies that depend on agriculture for their survival and growth. The following sections attempt to integrate our results and the available sources of information on environmental change, cross-cultural and socio-economic relations in the Bronze and Iron Age Near East in order to better understand the processes of regional niche construction and changes in olive and grape production.

Traditional land use patterns in the Levant of the 19^th^ and 20^th^ centuries indicate domination of cereal, vegetable crops and olive cultivation. Viticulture recedes into the background in terms of area [77,78]. It should be noted, however, that ancient vineyards probably occupied different areas in the past than they do today, many of which may have disappeared for religious reasons, as Dalman notes that in western Samaria (Nablus region) it is sometimes possible to see vineyards where there are now forests [79]. Similarly, ancient wine presses are often found in places where grapes are not grown today, which is related to the fact that Islam forbade the cultivation of grapes [79]. This may be an indication that wine production was more extensive in the past than it is today [133].

When we think of olive oil production centers, we usually associate them with surplus production that achieves high yields, which in semi-arid regions can only be achieved with good or surplus water availability, i.e., 600–800 mm (https://www.fao.org/land-water/databases-and-software/crop-information/olive/en/). For our samples, only a few sites fall into the 600 mm RAP or above. These are Tel Kabri in Israel and Tell Fadous-Kfarabida in Lebanon, which could be considered natural centers of high olive production. The sites between 400–600 mm RAP could theoretically be transformed into high production centers by irrigation. These include Tell Tweini, Tell Burak, and Jaffa, along the Levantine coast, as well as Hazor, Tel Burna, Tell Tayinat, Tell Atchana and Zincirli further inland. These sites fall chronologically mainly into the Iron Age or Middle to Late Bronze Age. Early Bronze Age sites, if not located in a natural center of high production, such as Tell Fadous-Kfarabida, are mainly found in the zone below 400 mm RAP. These include Hirbet ez-Zeraqon in Jordan and Tel Lachish in Israel, as well as Emar and Qatna in Syria, the latter also including also later settlement periods. From an economic point of view, these Early Bronze Age sites may have been self-sustaining in terms of olive production.

The southern Levantine EB I (ca. 3600−3000 BC) has been described as a transformative phase from settlements to agglomerated village societies, leading to contrasting settlement patterns in EBII that are comparable to urban structures, with full-fledged urbanism appearing only in EBIII (ca. 2800−2400 BC; [134]. Developments in the northern Levant are somewhat different, and the Early Bronze Age in northern Mesopotamia (ca. 3100−2000 BC) has been described as a period of ruralization and specialized small-scale economies that were nevertheless integrated into a larger economic network [135]. After 2600 BC, these developed into single larger city-states based on agricultural surplus production. The settlement development in the EB IV of Syria (2450−2000 BC) is similar to the Intermediate Bronze Age (ca. 2400−2000) in the southern Levant. The latter experienced a prolonged period of de-urbanization [135], while some settlements in the Khabur region were abandoned around 2200 BC, a process that many have linked to the 4.2 kyrs BP event (e.g., [109,136]. According to [136], the renewed climate change around 3.9 kyr BP would be characterized by increased sedentarism, political state formation, increased and improved surplus agricultural production, and politico-territorial expansion. From an emic perspective, we can classify all these actions as part of agricultural niche construction, but they were likely to have been locally highly variable, depending on regional and societal differences.

Against this background, the comparatively long period of the Early Bronze Age must be seen as an extremely dynamic situation for the establishment of a productive fruit tree culture. The southern Levantine archaeological record of olive and grape presses cut into the rock, usually located further away from settlements, documents an intensification of olive cultivation from Early Bronze Age I-III. Although the Early Bronze Age facilities are often indistinguishable as to whether they were used for olive oil or wine production, the technology changed over time or varied from region to region [137]. Our recent study argues that the increase in olives and grapes through time is evidence of favorable ecological and/or socio-economic conditions that stimulated and/or followed population growth in the 4th millennium BC and further promoted social stratification and trade [11]. Archaeologists generally report the increasing number of olive and grape presses from the Late Chalcolithic to the Roman period and pottery designed for the transport of liquids [3,14,138]. For the Early Bronze Age IV and the Middle Bronze Age, there is relatively little archaeological evidence of olive oil production, but this is probably partly due to the state of archaeobotanical research.

Inner-political change in Mesopotamia between c. 2000−1500 BC led to renewed political stability of major centers such as Mari and Ebla and increase in settlement sizes in the southern Levant included further agricultural development and expanded trade in products such as olive oil and wine [135, 139]. One of the most important finds of Bronze Age wine production comes from the Middle Bronze Age site of Tel Kabri. In the storage wing of the Middle Bronze Age palace (c. 1800−1700 BC), 150 pithoi were excavated, 40 of which were large storage vessels that were analyzed for their organic content using gas chromatography-mass spectrometry (GC-MS). These revealed the presence of wine to which honey, storax and terebinth resin, and cedar oil had been added [6,140]. The excavators estimate the total capacity of the storage vessels in this wing to be over 20,000 liters. This underscores the enormous cultural importance of wine production in the Middle Bronze Age, which is further supported by Δ^13^C values at Tel Kabri and other Middle Bronze Age sites indicating low stress signals in grape pips, despite sometimes low RAP, most likely due to irrigated vines in numerous areas during this period.

Based on the Δ^13^C threshold of below 15.5 ± 0.5‰ proposed by [61] as indicative of severe drought stress, there should have been particularly dry conditions at Lachish, Qatna, Tell Tweini, and Tell Atchana around the Middle to Late Bronze Age transition. However, increased aridity around this time has been discussed only incidentally in the palaeoclimate literature [102,103], and apart from the continuous Holocene drying, other factors may have played a role in these coincidences of poorly watered olive groves. These could be related to the significant political realignment in the Near East at the transition from the Middle to the Late Bronze Age, at least in some regions. The end of the Pax Amoritica, brought about by the campaigns of the Hittite and Egyptian empires, is reflected in the level of destruction at many settlements in the Levant, including Alalakh (Tell Atchana) and others. Among the characteristics of this period are political fragmentation and increasing competition between states [141]. Such developments can affect agricultural landscapes as much as climate change. The withdrawal of agricultural labor to maintain state power structures during periods of unrest as well as a loss of access to local and regional markets that probably lowered demand for olives/grapes, may have led to poor management of olive groves in some areas, as may have been the case in the Levant.

With the beginning of the Late Bronze Age (c. 1550 BCE), Egypt began to build its Levantine empire by periodically using military force to extort annual tribute from city-states. Various armed conflicts between Egypt, its northern vassals, and Levantine kingdoms, are documented in the Amarna Letters for the Late Bronze Age IIA (1400−1300 BC), as well as in the Hittite Annals. Constant turmoil, sieges, rebellions, and occasional peace treaties between different groups of Syria, the Hittite Empire and Egypt may have had an impact on agricultural production in these regions [135,139].. During the Levantine LB IIB (1300−1200 BC) to the end of Iron IA, Egyptian infiltration is present in ritual and religious behavior, as well as in exports, including cultic and luxury items, raw materials, food, and agricultural products such as catfish and grain [139]. Δ^13^C values of Late Bronze Age grapevines indicate a considerable general drought stress (Fig 10) which also applies to olive trees (Fig 9) and fruits, although many of the sites are situated above the 400 mm RAP (Fig 5c). At some sites this also applies to barley (Fig 6). This can be reconciled with the unrest and military conflicts described above and likely associated collapsing of agricultural production systems.

Considering the archaeological evidence for oil and wine production during the Late Bronze Age, the widespread use of the so-called Canaanite jars throughout the Levant and beyond is associated with an extensive exchange of agricultural products. Beginning in the Late Bronze Age II, new types of pressing facilities are associated with an increase in production to meet the high demand for olive oil in Egypt [142], which continued into the Iron Age I (at least in the 12th century BC).

In the southern Levant, in addition to rock-cut installations, walled and free-standing commercial wine presses also existed, especially in later periods, which have probably been underrepresented in the archaeological record for conservation reasons [3]. The few examples of plastered wine presses from the Bronze Age are mainly from the Late Bronze Age IIA-B (c. 14th-13th centuries BC) at Aphek, west of Palace IV on the edge of the Acropolis [143]. Since places in the southern Levant were under strong Egyptian influence in the Late Bronze Age, the expansion of wine production from the mountainous interior to the coastal plain can be linked to Egyptian sovereignty over the southern Levant and the transportation of wine to Egypt.

A relatively dry sequence between 1200 and 900 BC (3150−2900 cal BP) has been linked to crop failure, political upheaval, and economic collapse, which together determine social stress and famine [103,115,144,145]. In addition, around 1200 BC, migrations and invasions are widely recorded throughout the Mediterranean, involving many different cultural groups usually referred to as “Sea Peoples”, a phenomenon that can be considered as a complex migratory conflict over the entire Eastern Mediterranean and adjacent regions [146], with enormous impact on the ancient economy and culture. Social upheavals and epidemics probably accompanied these developments [117,147]. Some northern Mesopotamian centers disintegrate with the end of the Late Bronze Age, and the Levantine economy was hit by grain shortages, as evidenced by texts from Hittite Anatolia that mention “years of famine” [147]. The Hittite king Hattušili III appealed to Pharaoh Ramses II for help, since Egypt maintained an agriculture that was not dependent on rainfall, and although, according to the letters, Egypt sent ships with grain to Hatti, the appeals continued until the 11th century BC, when Egypt had lost its strong position in the southern Levant. Ugarit had also lost its economic and political role, causing surrounding settlements such as Tell Tweini to also decline [148]. The decline of agriculture probably had multiple causes, and its role may have been very different at different sites. Accordingly, archaeobotanical results and stable isotope data must be considered in combination for individual sites. This combination of data is still very patchy, but our datasets provide initial clues to disentangle the effects of environmental and politically motivated threats and their impact on societal collapse.

In the northern Levant a long tradition of olive oil production at Ugarit is reflected in the very diverse installations for olive processing [149]. Textual finds from the royal archives of the site also document the great economic importance of olive oil, which was not only produced in various sectors of the kingdom, but was also used as a means of payment in the tax system [150] and, in turn, as a means of payment for the wages of royal employees [151] and determined the economic priorities within the kingdom [10,148]. Ugarit and neighbouring Tell Tweini were integral parts of the extensive trade networks in the eastern Mediterranean and the geographical proximity of both cities and their access to various resources probably led to economic cooperation and mutual benefit from the flourishing trade of the time. This may be one of the reasons why the olive and grape cultivation at Tell Tweini stands out from other sites investigated here due to the exceptionally careful management of the plantations [10]. The good water supply of the plantations partially related to the favorable natural location probably accounts for the relatively low drought stress around 1100 BCE (S1 and S2 Figs; [10] and somehow contrasts the climatic evidence [110]. Nevertheless, both settlements declined at the end of the Bronze Age in relation with the political unrests and collapsing commerce.

The Iron Age IA (1200−1150 BC) continues as a period of disintegration of eastern Mediterranean polities and population migration. Egyptian influence in the Levant further declines, but it retains some control over the region, as indicated by archaeological evidence at several ancient cities (e.g., Lachish, Beth Shean, Deir el-Balah, Megiddo, Alalakh; [139]. During the early Iron IB (1150−1000 BC), Egyptian state-level contact with the eastern periphery of Philistia and southern Judea was minimal. Textual evidence is scarce and difficult to assess for credibility [139]. The Iron Age IIA (1000−925 BC) spans the biblical period of Israel’s emergence and dominance over Philistia, Edom, Moab, Ammon, and Damascus. The 9th century BC is also marked by the beginning of the Neo-Assyrian period. Assyrian interest in the Levant coincides with the emergence of new Iron Age polities following the crisis and resulting upheavals that characterized the end of the Late Bronze Age [cf. 140]. Territories west of the Khabur and north of the Euphrates, previously conquered during the Middle Assyrian period, were attacked by Neo-Assyrian kings who considered these regions still to be part of Assyria [152]. Assyrian interest in the region was often related to agricultural productivity [153]. In conjunction with Assyria’s westward expansion in Iron IIB (925−700 BC), Egypt changed its attitude toward its Levantine neighbors and returns to more diplomatic relations with Israel [139]. In the following centuries, Assyria dominates Palestine, and Tiglath-pileser III invades the Levantine coast and establishes a “guard” on Egypt’s eastern frontier by 732 BC [139]. Assyrian interest in the region has often been linked to agricultural productivity, but direct records based on archaeobotanical remains are problematic when it comes to linking them to the economic interests of other cultural groups [154]. Faust [155] argues, that for the specific example of Ekron, the often-claimed Assyrian interest in olive oil is not supported by the archaeological evidence, which rather shows that prosperity in the southern Levant was always outside the borders of the Assyrian Empire, and whenever a new prosperous region was conquered by the Assyrians, prosperity ceased.

As indicated by the Δ^13^C values, most of our Iron Age grape samples are associated with settlements above 500 mm RAP, in contrast to earlier periods. In relation to targeted surplus production, Iron Age viticulture seems to have been situated in more favorable rainfall patterns. The same is true for Iron Age olive cultivation, which was primarily located in regions with more than 400 mm RAP, whereas cultivation in earlier periods seems to have been confronted with more or less suitable rainfall ranges. However, comparing the more diffuse patterns, close to the 500 mm RAP limit, of Iron Age vines (Fig 4C) with those of olives (Fig 5C), we could conclude that while vines were cultivated even in regions that were naturally marginal and required a higher labor input with occasional irrigation, olives were a kind of economic background feature, i.e., an agronomic component that was not as intensively forced to fit into all kinds of agricultural systems as it was the case for grapes, similar to what is suggested by the ethnographic record. In other words, our results indicate a more or less strong manipulation of the niches suitable for cultivation. According to our results, Iron Age viticulture reflects the strongest form of niche construction. This corroborates the settlement distribution with grape evidence in the archaeobotanical record. At the time of the incorporation of much of the Levant into the Neo-Assyrian Empire (9th-8th century BC) an expansion of settlements with grape evidence to the east has been detected [11]. The situation in the Levant documents a peak in the cultivation of olives and grapes, possibly oriented to maritime trade.

The well-organized production of fruit tree products is further supported by the archaeological record of oil and wine production equipment and installations, which became increasingly complex over time and also show regional differences [137]. Iron Age finds of plastered wine presses are known from a number of sites [8,156–160]. The location of many of the wine presses near the coast suggests that wine production in the southern Levant was at least partly intended for Mediterranean trade [8]. The Mediterranean wine trade experienced a significant upswing in the Iron Age II. The evidence from Ashkelon, Tel Michal, and Tell el-Burak supports the theory that wine was produced on a large scale in the brick and plaster installations of Iron Age II and that this method of production was linked to the local royal courts and elites [8]. This is very much in line with the Iron Age Δ^13^C values of grape seeds, which generally show lower stress signals compared to the Late Bronze Age (Fig 4C), which is particularly evident at sites in the southern Levant (Fig 7).

The lever principle as a technological extension for the pressing process was introduced in the southern Levant during Iron Age I (e.g., Tel Dan), although most of these lever-weight presses are documented for Iron Age II, which took place within the framework of the Iron Age II territorial states, first in the north and then in the south of the southern Levant [3,137,161]. The most impressive example is Ekron, where 115 installations for oil production were excavated in production buildings along the inner fortification wall, covering 20–30% of the city area [14,157]. This corresponds to a calculated production capacity of 245,000 liters and indicates a flourishing of the olive oil trade in Iron Age II [162,163], which went far beyond all previously attested forms of surplus production and is therefore often referred to as “industrial production” in the context of the Neo-Assyrian economic system. A close connection between the upswing in olive oil production as early as the 8^th^/7^th^ centuries and the increase in population in Judah in the course of the conquest of Israel and Ephraim by the Assyrians is interpreted by Zwickel [164] from biblical text sources. According to Faust [155], the 7th cent. BC, the southern Levant is characterized as a period of economic prosperity attributed to Assyrian rule (the so-called “Pax Assyriaca”) and ended by the Babylonian campaigns at the end of the century. Based on settlement patterns, archaeobiological remains, and a review of the research literature, they argue “that the economies of both Judah and Philistia were well integrated into the larger economic system of the period and should be seen as almost one economic unit (or region) within that system, the driving force behind which was the Phoenician maritime trade. The ancient Hebrew inscriptions, such as the 80 ostraca of the 8th century BC from Samaria, provide economic texts that are interpreted as lists of oil and wine deliveries to the palace, either as taxes or as a one-time levy or as deliveries from royal lands [165]. The mention of both wine and oil is always accompanied by an indication of quality, thus suggesting the existence and application of a wide range of quality characteristics and possibly even the cultivation of different grape and olive varieties. On the other hand, the inscription on the inside of a round jar base from Tell el-Qasile in the Middle Coastal Plain, whose botanical findings did not yield any olive remains, is particularly revealing for the question of the importance of the trade areas. The writing represents a delivery note or invoice for presumably 1100 jars of oil and leads the authors to the conclusion that the oil was produced in Palestine and then shipped from Tell el-Qasile to Phoenicia, Egypt or another region of the Mediterranean [165].

By the end of the 7th century BC, it is apparent that some of the oil production centers, such as Ekron, were losing their function.

Considering the quality of agricultural niches for the sites we examined, the obvious importance of local precipitation conditions comes to the fore. Most of the sites we analyzed are located in regions with sufficient precipitation for rainfed cultivation of these tree crops (Table 1), which means that irrigation measures appear to have been necessary only to a limited extent. The most important production centres, which are also characterised by intensive cultivation in terms of archaeobotany, coincide with these localities, whereas where irrigation was necessary, the particular archaeobotanical crop abundances tend to indicate, at least in part, minor production (Table 1).

**Table 1:** Agricultural niche qualities for grape and olive cultivation based on the samples we analyzed for this study, reconstructed rainfall, drought stress signals and estimated scope of irrigation. Minus in the columns of ‘indication of irrigation’ refers to incomplete data.

	Reconstructed rainfall suitable for rainfed olive >300 mm	Rainfall suitable for rainfed grape >400 mm	Mean stress signal based on Δ^13^C values in fruit remains (O = olive, V = grape)	Estimate of scale of olive (O) and grape (V) cultivation based on archabotany (Deckers et al. 2024)	Indication for irrigation of olive based on archaeobotany and stable isotope evidence	Indication for irrigation of grape based on archaeobotany and stable isotope evidence
Atchana_LBA	yes	yes	O: moderate-high V: low	O: low, V: medium	no	no
Burak_IA	yes	yes	O: low, V: no stress	O: large, V: large	no	no
Burna_IA	yes	yes	O: high	O: medium, V: medium	no	–
Emar_EBA	no	no	O: moderate	O: low, V: low	yes	–
Emar_MBA	no	no	O: high	O: none, V: medium	possible	–
Emar_LBA	no	no	O: no stress	O: low, V: medium	yes	–
Fadous_EBA	yes	yes	O: no stress, V: no stress	O: large, V: medium	no	no
Jaffa_IA	yes	yes	O: low, V: moderate	O: medium, V: medium	no	no
Kinet_EBA (late)	yes	yes	V: no stress	O: medium, V: none	–	no
Lachish_MBA	yes	yes	O: moderate, V: no stress	O: large, V: low	no	no
Lachish_LBA	yes	yes	O: moderate, V: high	O: large, V: none	no	no
Mozan_EBA	yes	no	V: moderate	O: none, V: medium	–	possible
Mozan_MBA	yes	no	V: low	O: low or imported, V: low	–	yes
Qatna_EBA	no	no	O: high, V: moderate	O: low, V: large	possible	yes
Qatna_MBA	no	no	O: moderate V: low	O: low, V: large	possible	yes
Qatna_LBA	no	no	O: moderate, V: moderate	O: low, V: medium	yes	yes
Tweini_MBA	yes	yes	O: low-moderate, V: moderate-low	O: large, V: large	no	no
Tweini_LBA	yes	yes	O: no stress, V: no stress	O: large, V: large	no	no
Tweini_IA_after 1100	yes	yes	O: no stress, V: moderate	O: large, V: large	no	no
Zeraqon_EBA	yes	no	O: high-moderate, V: high-moderate	O: large, V: large	possible	possible
Zincirli_IA	yes	yes	O: low, V: low,	O: low, V: low	no	no

## Conclusions

Stable carbon isotope analysis of more than 1,500 charred olive and grape remains from archaeological sites in the Levant and northern Mesopotamia and their interpolation provided some important insight into Bronze and Iron Age production patterns.

The main trends in our data include a significant accumulation of Iron Age sites in olive-growing and wine-producing regions above 500 mm RAP. However, there are different trends for the two tree crops. Diachronic differences show that viticulture exhibits irregular stress behaviour with regard to both the trees and their fruits, while olive cultures are relatively stable, and the stress levels of trees and fruits are balanced, which appears to be related to the species-specific differences in physiological and agronomic characteristics. In the case of vines, increasing stress is evident in the plants from the Early Bronze Age to the end of the Late Bronze Age, followed by a subsequent decrease in stress. In contrast, the stress levels in the grapes fluctuate considerably, with higher stress levels in the Late and Early Bronze Age and lower stress during the Middle Bronze Age and Iron Age. Despite the relatively constant accumulative stress levels in the olive, the greatest stress is recognisable in the Early Bronze Age.

In addition, there are indications of increased stress during the two climate events around 4.2 kyr BP and 3.2 kyr BP at a few sites, while at other sites the stress is lower than expected. Interestingly, a relatively high stress signal is more likely to be detected at sites in regions with higher precipitation, while sites in regions with lower precipitation show lower than expected stress signals. This phenomenon is most likely to be explained by the selective practice of irrigation in generally drier regions.

While a differentiation between irrigated and non-irrigated crops can be made by comparing annual signals (wood) and seasonal signals (fruits), interpolations of Δ^13^C values can map isoscapes of water availability for plants within a defined geographical region. The basic assumptions are that (1) annual precipitation is higher than seasonal precipitation, (2) water deficits are reflected in a consistent relationship between the vegetative and reproductive organs within a taxon. This means that a higher stress signal, i.e., a lower Δ^13^C value is to be expected in the fruits of a taxon than in the wood, provided that no seasonal irrigation has been carried out to maintain high yields. If, on the other hand, the Δ^13^C values of the fruits are only slightly lower, the same or higher than in the wood, irrigation becomes very likely. This connection can often be seen even more clearly in a diachronic comparison of the interpolation maps. The interpolations also confirm the physiological differences between the crop species. For example, the grapevines in the Syrian interior (Tell Mozan) and Jordan (Hirbet ez-Zeraqon) show comparatively higher stress signals than the barley crops. This approach also allows us to recognise agricultural practices focused on producing surplus for trade. At coastal sitessuch as Tell Tweini, vineyards were cultivated with greater care in terms of water supply than barley, suggesting an emphasis on grape production for export. As far as water availability and the general lack of irrigation are concerned, the cultivation of olives followed a similar pattern to barley cultivation, at least during the Early Bronze Age.

Overall, however, there is still a great diversity in water availability at the individual sites, and early farmers apparently took into account various aspects such as the dietary value of the product and its economic profit, as well as ecological factors and weather conditions at the location, which they weighed against each other, generating the highly site-specific diversity in the individual parameters examined here. This adds to our understanding of agricultural niche construction, which indicates that grapevine was cultivated even in regions that were ecologically marginal and required a higher labor input. Such manipulation of niches is particularly evident for Iron Age viticulture.

## Supporting information

S1 Fig Δ^13^C values of olive (green) and grape (violet) from different settlement phases (with ca. years in BC) at different locations. The blue line marks a possible threshold of drought stress for grape, indicated by values below this line of 19.2‰ in orientation to the Ehrlich threshold at 15.5 ± 0.5‰ [61] for olive fruit: 1 Lachish_1600, 2 Lachish_1600, 3 Lachish_1470, 4 Lachish_1470, 5 Olive_Lachish_1380, 6 Lachish_1380, 7 Lachish_1300, 8 Lachish_1300, 9 Lachish_modern, 10 Burna_780, 11 Gal’on_modern, 12 Jaffa_1130, 13 Jaffa_1130, 14 Pella_stone_modern, 15 Zeraq_3070, 16 Zeraq_3070, 17 Zeraq_2970/40, 18 Zeraq_2970, 19 Zeraq_2950, 20 Zeraq_2870, 21 Zeraq_2870, 22 Keisan_modern, 23 Kabri_1800, 24 Kabri_1700, 25 Kabri_1700, 26 Kabri_modern, 27 Hazor_1450, 28 Hazor_1350, 29 Hazor_1200, 30 Hazor_950, 31 Hazor_750, 32 Hazor_700, 33 Burak_660, 34 Burak_660, 35 Burak_550, 36 Burak_550, 37 Burak_450, 38 Burak_450, 39 Jbeil_stone_modern, 40 Fadous_2850, 41 Fadous_2770, 42 Fadous_2650, 43 Fadous_2651, 44 Fadous_2550, 45 Fadous_2550, 46 Fadous_1850, 47 Basbina_stone_modern, 48 Qatna_2550, 49 Qatna_2550, 50 Qatna_2100, 51 Qatna_2000, 52 Qatna_2000, 53 Qatna_1950, 54 Qatna_1800, 55 Qatna_1420, 56 Qatna_1400, 57 Qatna_1150, 58 Qatna_600, 59 Qatna_cf_modern, 60 Tweini_2200, 61 Tweini_2200, 62 Tweini_1900, 63 Tweini_1900, 64 Tweini_1800, 65 Tweini_1700, 66 Tweini_1700, 67 Tweini_1600, 68 Tweini_1600, 69 Tweini_1400, 70 Tweini_1400, 71 Tweini_1300, 72 Tweini_1300, 73 Tweini_1100, 74 Tweini_1100, 75 Tweini_600, 76 Emar_2750, 77 Emar_1850, 78 Emar_1400, 79 Atchana_1800, 80 Atchana_1500, 81 Atchana_1380, 82 Atchana_1258, 83 Atchana_1200, 84 Tayinat_2400, 85 Tayinat_2400, 86 Tayinat_2300, 87 Tayinat_2300, 88 Tayinat_2180, 89 Tayinat_2180, 90 Tayinat_1200, 91 Tayinat_1100, 92 Tayinat_980, 93 Tayinat_980, 94 Tayinat_750, 95 Tayinat_710, 96 Tayinat_670, 97 Taynat_modern, 98 Halaf_460, 99 Kinet_2200, 100 Mozan_2500, 101 Mozan_2100, 102 Mozan_1925, 103 Zincirli_825, 104 Zincirli_800, 105 Zincirli_800, 106 Zincirli_750, 107 Kumtepe B3_3100, 108 Troy_3000, 109 Troia_2100, 110 Troia_1500, 111 Troia_1240, 112 Troia_440.
(TIF)

S2 Fig Δ^13^C values of olive stones (full color) and wood charcoal (dark grey) from archaeological sites, and some modern specimens (red lined; L = leaves, P = pulp, S = stones). The orange line indicates the Δ^13^C threshold for olive fruit under severe drought stress at below 15.5 ± 0.5‰as defined by Ehrlich [61]. The numbers refer to the different settlement phases (with ca. years in BC) at the different locations (C_ = wood charcoal): 1 Olea_Lachish_1600, 2 Olea C_Lachish_1595, 3 Olea_Lachish_1470, 4 Olea C_Lachish_1457, 5 Olive_Lachish_1380, 6 Olea C_Lachish_1370, 7 Olea_Lachish_1300, 8 Olea C_Lachish_1250, 9 Olea_Lachish_modern, 10 Olea_Burna_780, 11 Olea_Gal’on_modern, 12 Olea_Jaffa_1130, 13 Olea_Pella_leaves_modern, 14 Olea_Pella_pulp_modern, 15 Olea_Pella_stone_modern, 16 Olea_Zeraq_3070, 17 Olea C_Zeraqon_3000, 18 Olea_Zeraq_2970, 19 Olea_Zeraq_2940, 20 Olea_Zeraq_2870, 21 Olea C_Zeraqon_2800, 22 Olea C_Zeraqon_2700, 23 Olea_Keisan_modern, 24 Olea_Kabri_1800, 25 Olea_Kabri_1700, 26 Olea_Kabri_modern, 27 Olea_Hazor_1200, 28 Olea_Hazor_700, 29 Olea C_Burak_1800, 30 Olea C_Burak_1400, 31 Olea_Burak_660, 32 Olea C_Burak_660, 33 Olea_Burak_550, 34 Olea C_Burak_550, 35 Olea_Burak_450, 36 Olea C_Burak_450, 37 Olea_Jbeil_leaves_modern, 38 Olea_Jbeil_pulp_modern, 39 Olea_Jbeil_stone_modern, 40 Olea C_Fadous_2900, 41 Olea_Fadous_2850, 42 Olea_Fadous_2770, 43 Olea C_Fadous_2700, 44 Olea_Fadous_2650, 45 Olea_Fadous_2550, 46 Olea C_Fadous_2550, 47 Olea_Fadous_1850, 48 Olea C_Fadous_1800, 49 Olea_Basbina_stone_modern, 50 Olea_Basbina_pulp_modern, 51 Olea_Koubba_leaves_modern, 52 Olea_Koubba_pulp_modern, 53 Olea_Qatna_2550, 54 Olea C_Qatna_2375, 55 Olea_Qatna_2100, 56 Olea C_Qatna_2125, 57 Olea_Qatna_2000, 58 Olea C_Qatna_1900, 59 Olea_Qatna_1800, 60 Olea_Qatna_1420, 61 Olea C_Qatna_1420, 62 Olea_Qatna_1400, 63 Olea_Qatna_1150, 64 Olea_Tweini_2200, 65 Olea_Tweini_1900, 66 Olea_Tweini_1700, 67 Olea_Tweini_1600, 68 Olea_Tweini_1400, 69 Olea_Tweini_1300, 70 Olea_Tweini_1100, 71 Olea_Tweini_600, 72 Olea_Emar_2250, 73 Olea_Emar_1850, 74 Olea_Emar_1400, 75 Olea C_Jerablus_2475, 76 Olea C_Mozan_1800, 77 Olea C_Kinet_1775, 78 Olea_Kinet_1340, 79 Olea C_Kinet_1025, 80 Olea C_Kinet_775, 81 Olea C_Kinet_625, 82 Olea C_Kinet_550, 83 Olea C_Kinet_475, 84 Olea C_Kinet_202, 85 Olea C_Kinet_2335, 86 Olea_Atchana_1800, 87 Olea_Atchana_1500, 88 Olea_Atchana_1425, 89 Olea_Atchana_1387, 90 Olea_Atchana_1380, 91 Olea_Atchana_1342, 92 Olea_Atchana_1329, 93 Olea_Atchana_1258, 94 Olea_Atchana_1240, 95 Olea_Tayinat_2400, 96 Olea_Tayinat_2300, 97 Olea_Tayinat_2180, 98 Olea_Tayinat_980, 99 Olea_Tayinat_750, 100 Olea_Tayinat_670, 101 Olea_Zincirli_800, 102 Olea C_Zincirli_2250, 103 Olea C_Zincirli_1800, 104 Olea C_Zincirli_1650, 105 Olea C_Zincirli_875, 106 Olea C_Zincirli_775, 107 Olea C_Zincirli_710, 108 Olea_Troia_440, 109 Olea C_Havat Hanania_1961.
(TIF)

S3 Fig PCA on mean, minimum and maximum Δ13C values as functional traits of the different sites and the reconstructed average precipitation (RAP) as environmental variables. (TIF)

S1 File Supplementary methods. (PDF)

S2 File Stable isotope data. Excel table of raw stable isotope data of seeds and wood charcoal.
(XLSX)

S3 File Reconstructed Annual Precipitation. (XLSX)
