# Supplementary figures and images for: Fluctuations of viti- and oleiculture traditions in the Bronze and Iron Age Levant
Source: PLoS One. 2025 Sep 17;20(9):e0330032. doi: 10.1371/journal.pone.0330032 (PMC12443296; doi:10.1371/journal.pone.0330032)

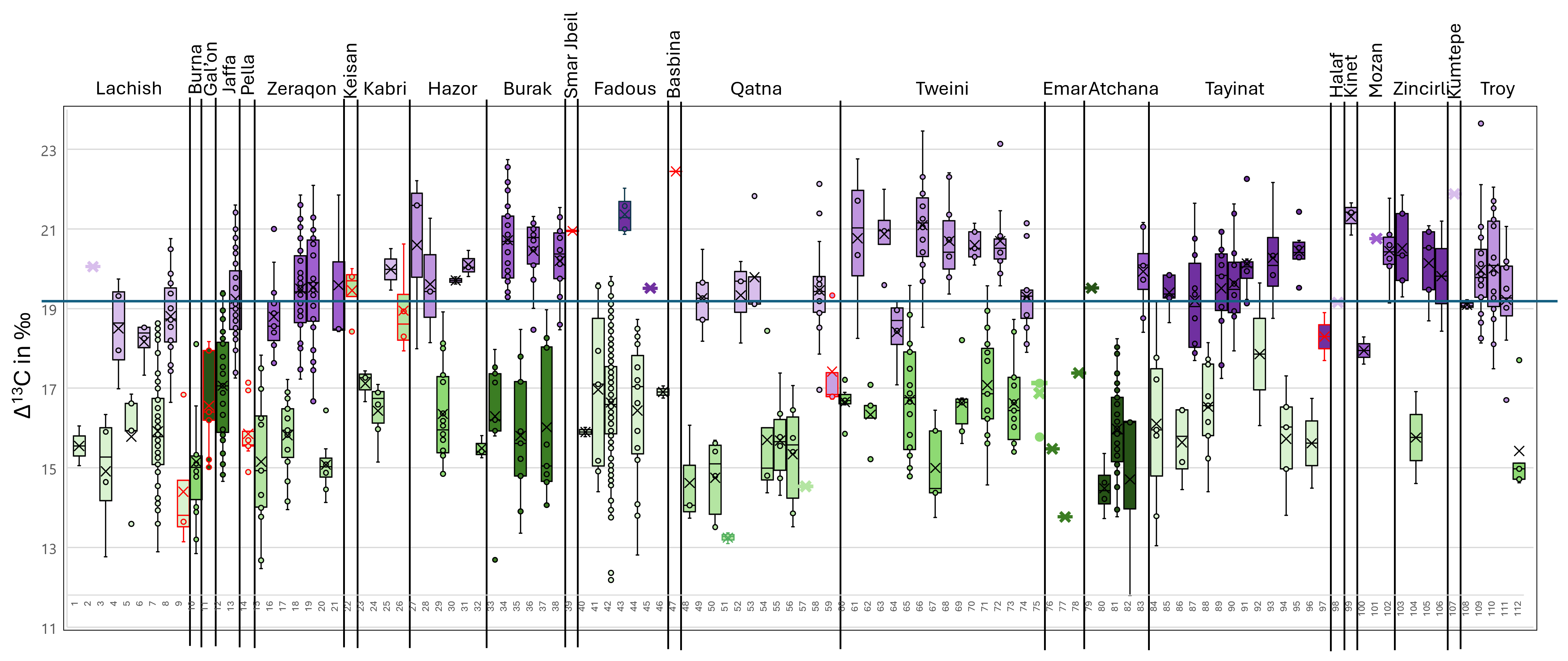

Supplement: S1 Fig — The blue line marks a possible threshold of drought stress for grape, indicated by values below this line of 19.2‰ in orientation to the Ehrlich threshold at 15.5 ± 0.5‰ [61] for olive fruit: 1 Lachish_1600, 2 Lachish_1600, 3 Lachish_1470, 4 Lachish_1470, 5 Olive_Lachish_1380, 6 Lachish_1380, 7 Lachish_1300, 8 Lachish_1300, 9 Lachish_modern, 10 Burna_780, 11 Gal’on_modern, 12 Jaffa_1130, 13 Jaffa_1130, 14 Pella_stone_modern, 15 Zeraq_3070, 16 Zeraq_3070, 17 Zeraq_2970/40, 18 Zeraq_2970, 19 Zeraq_2950, 20 Zeraq_2870, 21 Zeraq_2870, 22 Keisan_modern, 23 Kabri_1800, 24 Kabri_1700, 25 Kabri_1700, 26 Kabri_modern, 27 Hazor_1450, 28 Hazor_1350, 29 Hazor_1200, 30 Hazor_950, 31 Hazor_750, 32 Hazor_700, 33 Burak_660, 34 Burak_660, 35 Burak_550, 36 Burak_550, 37 Burak_450, 38 Burak_450, 39 Jbeil_stone_modern, 40 Fadous_2850, 41 Fadous_2770, 42 Fadous_2650, 43 Fadous_2651, 44 Fadous_2550, 45 Fadous_2550, 46 Fadous_1850, 47 Basbina_stone_modern, 48 Qatna_2550, 49 Qatna_2550, 50 Qatna_2100, 51 Qatna_2000, 52 Qatna_2000, 53 Qatna_1950, 54 Qatna_1800, 55 Qatna_1420, 56 Qatna_1400, 57 Qatna_1150, 58 Qatna_600, 59 Qatna_cf_modern, 60 Tweini_2200, 61 Tweini_2200, 62 Tweini_1900, 63 Tweini_1900, 64 Tweini_1800, 65 Tweini_1700, 66 Tweini_1700, 67 Tweini_1600, 68 Tweini_1600, 69 Tweini_1400, 70 Tweini_1400, 71 Tweini_1300, 72 Tweini_1300, 73 Tweini_1100, 74 Tweini_1100, 75 Tweini_600, 76 Emar_2750, 77 Emar_1850, 78 Emar_1400, 79 Atchana_1800, 80 Atchana_1500, 81 Atchana_1380, 82 Atchana_1258, 83 Atchana_1200, 84 Tayinat_2400, 85 Tayinat_2400, 86 Tayinat_2300, 87 Tayinat_2300, 88 Tayinat_2180, 89 Tayinat_2180, 90 Tayinat_1200, 91 Tayinat_1100, 92 Tayinat_980, 93 Tayinat_980, 94 Tayinat_750, 95 Tayinat_710, 96 Tayinat_670, 97 Taynat_modern, 98 Halaf_460, 99 Kinet_2200, 100 Mozan_2500, 101 Mozan_2100, 102 Mozan_1925, 103 Zincirli_825, 104 Zincirli_800, 105 Zincirli_800, 106 Zincirli_750, 107 Kumtepe B3_3100, 108 Troy_3000, 109 Troia_2100, 110 Troia_1500, 111 Troia_1240, 112 Troia_440. (TIF [file pone.0330032.s001.tif]

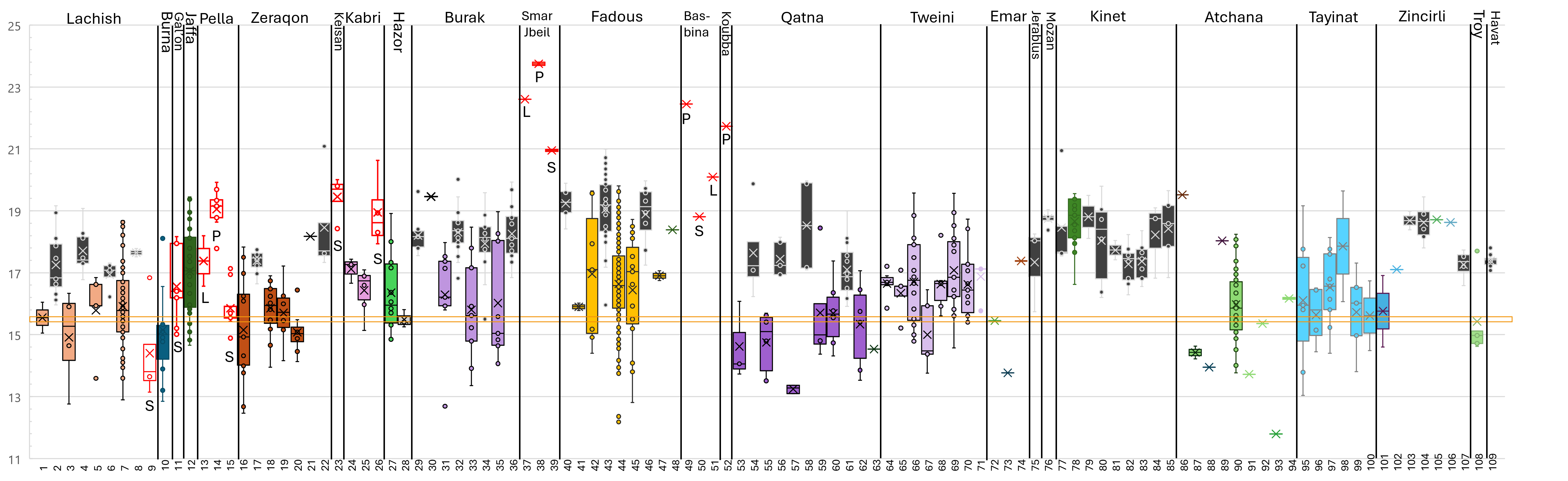

Supplement: S2 Fig — The orange line indicates the Δ13C threshold for olive fruit under severe drought stress at below 15.5 ± 0.5‰as defined by Ehrlich [61]. The numbers refer to the different settlement phases (with ca. years in BC) at the different locations (C_ = wood charcoal): 1 Olea_Lachish_1600, 2 Olea C_Lachish_1595, 3 Olea_Lachish_1470, 4 Olea C_Lachish_1457, 5 Olive_Lachish_1380, 6 Olea C_Lachish_1370, 7 Olea_Lachish_1300, 8 Olea C_Lachish_1250, 9 Olea_Lachish_modern, 10 Olea_Burna_780, 11 Olea_Gal’on_modern, 12 Olea_Jaffa_1130, 13 Olea_Pella_leaves_modern, 14 Olea_Pella_pulp_modern, 15 Olea_Pella_stone_modern, 16 Olea_Zeraq_3070, 17 Olea C_Zeraqon_3000, 18 Olea_Zeraq_2970, 19 Olea_Zeraq_2940, 20 Olea_Zeraq_2870, 21 Olea C_Zeraqon_2800, 22 Olea C_Zeraqon_2700, 23 Olea_Keisan_modern, 24 Olea_Kabri_1800, 25 Olea_Kabri_1700, 26 Olea_Kabri_modern, 27 Olea_Hazor_1200, 28 Olea_Hazor_700, 29 Olea C_Burak_1800, 30 Olea C_Burak_1400, 31 Olea_Burak_660, 32 Olea C_Burak_660, 33 Olea_Burak_550, 34 Olea C_Burak_550, 35 Olea_Burak_450, 36 Olea C_Burak_450, 37 Olea_Jbeil_leaves_modern, 38 Olea_Jbeil_pulp_modern, 39 Olea_Jbeil_stone_modern, 40 Olea C_Fadous_2900, 41 Olea_Fadous_2850, 42 Olea_Fadous_2770, 43 Olea C_Fadous_2700, 44 Olea_Fadous_2650, 45 Olea_Fadous_2550, 46 Olea C_Fadous_2550, 47 Olea_Fadous_1850, 48 Olea C_Fadous_1800, 49 Olea_Basbina_stone_modern, 50 Olea_Basbina_pulp_modern, 51 Olea_Koubba_leaves_modern, 52 Olea_Koubba_pulp_modern, 53 Olea_Qatna_2550, 54 Olea C_Qatna_2375, 55 Olea_Qatna_2100, 56 Olea C_Qatna_2125, 57 Olea_Qatna_2000, 58 Olea C_Qatna_1900, 59 Olea_Qatna_1800, 60 Olea_Qatna_1420, 61 Olea C_Qatna_1420, 62 Olea_Qatna_1400, 63 Olea_Qatna_1150, 64 Olea_Tweini_2200, 65 Olea_Tweini_1900, 66 Olea_Tweini_1700, 67 Olea_Tweini_1600, 68 Olea_Tweini_1400, 69 Olea_Tweini_1300, 70 Olea_Tweini_1100, 71 Olea_Tweini_600, 72 Olea_Emar_2250, 73 Olea_Emar_1850, 74 Olea_Emar_1400, 75 Olea C_Jerablus_2475, 76 Olea C_Mozan_1800, 77 Olea C_Kinet_1775, 78 Olea_Kinet_1340, 79 Olea C_Kin [file pone.0330032.s002.tif]

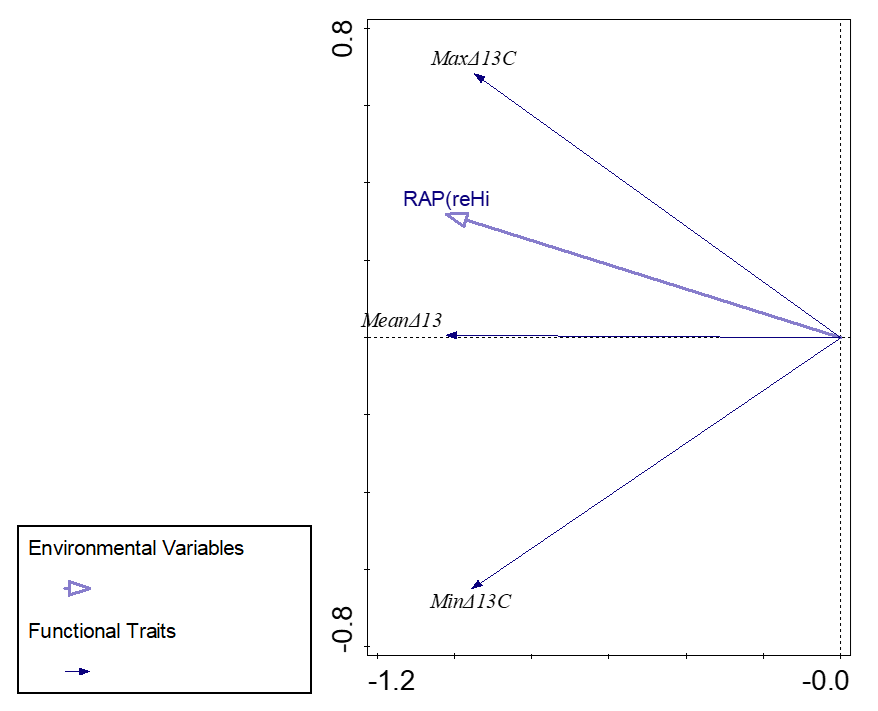

Supplement: S3 Fig — (TIF) [file pone.0330032.s003.tif]
