# Supplementary material for: Fluctuations of viti- and oleiculture traditions in the Bronze and Iron Age Levant
Source: PLoS One. 2025 Sep 17;20(9):e0330032. doi: 10.1371/journal.pone.0330032 (PMC12443296; doi:10.1371/journal.pone.0330032)
Supplement: S1 File — (PDF) [file pone.0330032.s004.pdf]

Supplementary method file

Stable carbon isotope analysis

Stable carbon isotope measurements were performed at SIBL using a Costech Elemental Analyser (ECS 4010) connected to a Thermo Scientific Delta V Advantage. Olive stones and grape pips from the Tell Tweini project were isotopically measured at the MPI Leipzig in an automated carbon and nitrogen analyzer (Carlo Erba) and analyzed for stable isotope ratios using a continuous-flow isotope ratio monitoring mass spectrometer (PDZ Europa Geo 20/20). The isotopic measurements, carried out at the Geochemistry Laboratory of the University of Tübingen, were performed using a FinniganMAT252 gas source mass spectrometer with a ThermoFinnigan GasBench II/CTC Combi-Pal autosampler.

Prior to the mass spectrometric measurements, all archaeobotanical specimens went through the following preparation steps: (1) removal of carbonates and acid soluble components in 1,8 ml 0.5 M hydrochloric acid (HCl) at room temperature for one hour, (2) rinsing of samples with ultrapure water, (3) humic acid removal with 0.2 % NaOH for 15 minutes, (4) rinsing of samples with ultrapure water, (5) acid wash with 0.5 M HCl at room temperature for one hour, (6) rinsing of samples with ultrapure water, (7) drying for 48 h at 50 °C. Stable isotopes ratios were reported in standard delta notation in parts per million (δ^{13}C ‰) that are calibrated against Vienna Pee Dee Belemnite (VPDB) using internationally calibrated standards. Errors were calculated using the average variation of the internal standard over at least one year of measurements and was less than 0.2‰.

Carbon concentration was generated from the stable isotope analysis using an internal standard (Glutamic Acid, 40.82 wt % carbon). Isotopic accuracy was monitored by routine analyses of in-house standards, which were stringently calibrated against international standards (e.g., IAEA-600, IAEA-CH-3, IAEA-CH-6, NBS 19, USGS24, USGS40). International and in-house standards are run daily and provided a linear range for δ^{13}C between –46 ‰ and +3 ‰. Analytical uncertainty in carbon isotope analysis was typically ±0.1 ‰ (2 s.d.) for replicate analyses of the international standards and ±0.2 ‰ (2 s.d.) on replicate sample analysis.

Data analysis

Since the Δ^{13}C measurements are not normally distributed (the p-value of a Shapiro-Wilk test was only 0.0016), we performed a non-parametric Friedman test, since we can expect Δ^{13}C values to depend on RAP based on plant physiological processes (e. g, [1-3]), and due to the fact that for some of the RAP values (reconstructed per site and chronology) we had more than one value (e.g., when Δ^{13}C values were available for grape and olive or seeds and charcoal from the same site), which would make a Kruskal-Wallis test less appropriate. Furthermore, since our data set for multivariate statistics used either mean or minimum Δ^{13}C values, the Friedman test is more appropriate as it takes into account within-subject variability, which can increase the sensitivity of the test to detect differences between conditions, whereas the Kruskal-Wallis test does not take into account within-subject variability. The p-value of 0.0001 in the Friedman test indicates a very strong statistical significance of the observed differences between the groups.

Since Δ^{13}C is primarily an indicator of plant water availability, the variance-covariance structure of the principal components, i.e. reconstructed precipitation (RAP) and Δ^{13}C measured in the different taxa and sites, was analyzed using principal component analysis (PCA). Both data types were entered as continuous

numerical data. Standardization and PCA were performed using Canoco 5 software [1]. Prior to these steps, Canonical Correspondence Analysis (CCA) was chosen to understand how the set of explanatory variables considered (RAP ranges, chronological ranges, elevation steps) relate to the mean $\Delta^{13}C$ values. The results are presented in ordination and attribute plots.

For CCA we used two datasets, with dataset 1 containing all $\Delta^{13}C$ variables, minimum, maximum and mean $\Delta^{13}C$ values (environmental variables) to allow analysis of samples for all three values, assuming that the variance of these values might also be important for particular environmental settings. Previous research suggests that a high range of variation in $\Delta^{13}C$ values within a sample often occurs in regions with probable irrigation [5]. Dataset 1 had to be reduced by targeting samples with more than two $\Delta^{13}C$ measurements per taxon, resulting in a reduction from 182 to 133 samples. Dataset 2 included RAP, elevation, and chronology, which were classified as landscape parameters. We decided not to use Redundancy Analysis (RDA) despite the fact that $\Delta^{13}C$ is compositional data and has a gradient of 0.2 SD units long. CCA can only handle quantitative explanatory variables, and if categorical variables need to be included, they must be converted into dummy variables. We omitted this step to take full advantage of the precise data available for RAP, elevation, and chronology instead of categories.

Interpolation

IDW is easy to implement and computationally efficient for small data sets. Its drawbacks are that it does not provide error estimates and assumes that the influence of points decreases uniformly with distance, which is not always realistic. Splines use piecewise polynomial functions to create a smooth surface and, like IDW, are deterministic. They can handle complex boundaries and varying data densities. The drawback is that they can overshoot, resulting in unrealistic values, especially at the edges of the data. It is computationally intensive and best suited for large data sets. Kriging is a geostatistical method that uses a weighted average of nearby data points, with weights derived from a model of spatial correlation (the variogram). Kriging takes into account not only the distance between points, but also the spatial arrangement and statistical properties of the data. The advantages are that it provides the best linear, unbiased prediction of the unsampled locations. Kriging also provides uncertainty estimates but is more complex and computationally intensive than IDW and splines.

In cases where the sample size is limited, IDW can be a more reliable method than more complex approaches such as kriging and splines. This is due to its deterministic nature and straightforward implementation, which make it less susceptible to the pitfalls associated with sparse data. Our analysis revealed that while kriging has the potential to produce realistic spatial predictions in terms of alignment with the general layout of the isohyets, it requires a larger dataset to capture meaningful patterns. We recognize that IDW does not quantify uncertainty, and our sparse sampling limits error assessment. However, our goal was to visualize broad trends, not precise predictions. Future work will consider uncertainty but also covariates with the use of mixed models to account for political, topographical or even cultural boundaries. The interpolated patterns suggest coherence in C13 dispersion, though this requires validation with denser sampling. Splines, on the other hand, resulted in distorted and likely unrealistic patterns, particularly for the distribution of Late Bronze Age grapes, due to the small sample size. Consequently, IDW was selected as the most suitable interpolation method, providing consistent and interpretable results given the constraints of our data.

References

1. Farquhar GD, Richards RA. Isotopic composition of plant carbon correlates with water-use efficiency of wheat genotypes. Australian Journal of Plant Physiology. 1984;11:539-52.

2. Ehleringer JR. Carbon and water relations in desert plants: an isotopic perspective. In: Ehleringer JR, Hall AE, Farquhar GD, editors. Stable isotopes and plant carbon-water relations. San Diego, CA: Academic Press; 1993. p. 155-72.

3. Araus JL, Ferrio JP, Voltas J, Aguilera M, Buxó R. Agronomic conditions and crop evolution in ancient Near East agriculture. Nat Commun. 2014;5. doi: 10.1038/ncomms4953.

4. Smilauer P, Leps J. Multivariate Analysis of Ecological Data Using CANOCO 5. second ed ed. Cambridge: Cambridge University Press; 2014.

5. Riehl S, Pustovoytov KE, Weippert H, Klett S, Hole F. Drought stress variability in ancient Near Eastern agricultural systems evidenced by δ13C in barley grain. Proceedings of the National Academy of Sciences. 2014;111(34):12348-53. doi: 10.1073/pnas.1409516111.
